# Dietary Responses in the Diabetic Brain: Transcriptomic Convergence and Comparison with an Alzheimer’s Disease Model

**DOI:** 10.3390/ijms27219957

**Published:** 2026-11-06

**Authors:** Bernard Kordas, Judyta K. Juranek

**Affiliations:** Department of Human Physiology and Pathophysiology, School of Medicine, Collegium Medicum, University of Warmia and Mazury, 10-082 Olsztyn, Poland; bernard.kordas@uwm.edu.pl

**Keywords:** diabetes mellitus, brain, intermittent fasting, ketogenic diet, Alzheimer’s disease, transcriptomics, gene expression profiling, pathway analysis, cross-species analysis

## Abstract

Dietary interventions alter brain gene expression in diabetes, but the extent to which their responses agree across experiments and reduce differences from controls remains unclear. Five public transcriptomic datasets were reanalyzed, spanning human cortical fractions, rat brain regions, diabetic mouse hippocampus with intermittent fasting (IF), diabetic whole brain with ketogenic diet, and APP/PS1 and wild-type hippocampus with IF. Gene and pathway comparisons assessed agreement across experiments. Statistical tests distinguished opposing disease and dietary effects from reductions in the remaining distance from controls. IF and ketogenic diet identified 6569 and 999 genes, respectively, in diabetic mice. Their effects showed modest agreement (Pearson *r* = 0.205), with 447 genes significant in both experiments, including 359 with matching directions. They shared 59 pathways with a matching direction at a fixed intergene correlation of 0.01; these overlaps were not supported when correlations were estimated from the data. IF responses in diabetic and APP/PS1 hippocampus agreed poorly (*r* = −0.056). No human genes met a per-contrast false discovery rate < 0.05. Thirteen diabetic genes showed significant opposition of disease and IF effects; neither primary fasting experiment demonstrated contraction toward controls. The results support limited agreement between the diabetic dietary experiments and little agreement in IF responses across the examined disease contexts. They do not establish transcriptomic restoration.

## 1. Introduction

Transcriptomic studies have described gene expression changes in diabetes in enriched human cortical fractions and distinct rat brain regions [1,2]. A study of mouse hippocampus also examined how intermittent fasting (IF) modifies these changes [3]. Comparing these datasets can help distinguish responses shared across experimental settings from those that depend on tissue, species, or intervention. Reported overlaps between diabetes and Alzheimer’s disease (AD), including genes involved in immune responses, provide a rationale for extending this comparison to AD [4,5]. Here, the AD model provides an additional test of whether the transcriptional responses to IF observed in diabetes extend to a different disease context.

The key question is whether dietary interventions produce a shared brain transcriptional response across diabetic experiments and whether that response is also observed in an AD model. Agreement across experiments would support testing of shared processes, while disagreement would identify limits to their transferability. This comparison does not assume that every expression change associated with disease is harmful or should be reversed. A dietary effect opposite to a disease effect does not necessarily bring expression closer to controls. It may reduce the difference, cross the control value, or overshoot beyond the original distance.

Two additional experiments extend this comparison. Ketogenic diet in db/db mice tests whether an IF response also occurs with another dietary intervention in diabetes [6]. The factorial APP/PS1 and wild-type experiment tests transfer of IF responses to AD and their dependence on the genotype [7]. Altered cerebral insulin and insulin-like growth factor signaling motivates comparison of brain changes in diabetes and AD [8,9]. Experimental fasting and ketogenic protocols have also supported neuronal resistance or memory in distinct mouse models [10,11], while time-restricted feeding altered hippocampal transcription and improved pathological or behavioral outcomes in AD models [12].

The study addressed three linked questions: how brain expression changes agree across diabetic datasets, whether IF and ketogenic diet share expression responses in diabetes, and whether IF responses agree between diabetic and APP/PS1 hippocampus. A further question was whether fasting effects oppose changes associated with disease and reduce the remaining distance from controls. Each experiment was analyzed separately before comparison across common genes and pathways.

## 2. Results

### 2.1. Gene Responses Differed Across Human Fractions and Rodent Models

No gene among the 20,848 tested per human fraction met a per-contrast FDR < 0.05 in primary or alternative covariate models. Removing both flagged astrocyte controls also yielded no discoveries, although rank correlation of moderated *t* statistics with the primary model decreased to 0.653, versus 0.989–0.998 for alternative covariate specifications. Neuronal omission of donor RH028 yielded one significant gene.

Applying the published human selection criteria [2] reproduced 2202 astrocyte, 1227 vascular, and 912 neuron probe-set discoveries at unadjusted *p* < 0.05 and a fold-change threshold of 1.2. None survived BH adjustment across all 54,675 probe sets per fraction.

STZ-versus-control comparisons identified 1778 genes in rat hippocampus, 145 in prefrontal cortex, and 19 in striatum; corresponding GK counts were 1905, 1187, and 244. Diabetic mouse hippocampus yielded 891 disease genes, 6569 IF genes, and 7103 residual-comparison genes. Figure 1 shows their direction and proportion within each tested universe.

Rat discovery counts depended on normalization: STZ hippocampus yielded 1778 genes with quantile normalization versus 171 with log_2_ alone, whereas GK striatum yielded 244 versus 979 (Appendix A Figure A1 and Appendix A Table A1). Higher and lower discoveries are shown separately for each primary contrast in Figure 1.

### 2.2. Pathway Evidence Depended on Correlation Assumptions

Human astrocyte-, vascular-, and neuron-enriched fractions yielded 1071, 81, and 90 CAMERA-PR discoveries at a fixed correlation of 0.01; matched counts from CAMERA applied to expression matrices were 1069, 81, and 91. At fixed 0.05, counts from CAMERA applied to expression matrices fell to 39, zero, and zero. Estimated correlations yielded none, with a minimum astrocyte FDR of 0.676.

CAMERA applied to expression matrices at fixed 0.01 identified 20 sets in GK hippocampus, 10 in STZ prefrontal cortex, and two in GK striatum; other rat contrasts yielded none. At fixed 0.05, no rat contrast retained discoveries. Mouse disease, IF, and residual contrasts yielded 86, 158, and 269 sets at fixed 0.01, falling to zero, three, and 28 at 0.05. Estimated correlations yielded none in any comparison. Figure 2 compares pathway discovery counts across the three correlation settings.

### 2.3. Translation and Respiratory Annotations Remained Exploratory

Astrocyte-enriched results at fixed 0.01 included upward ranks for translation, SRP targeting, the EIF2AK4/GCN2 response, and oxidative phosphorylation. Their estimated residual correlations were approximately 0.272, 0.443, 0.460, and 0.338, respectively; none remained significant with estimated correlations. Vascular results included overlapping translation annotations, whereas neuronal results included downward respiratory and ATP-coupled ranks.

RPL/RPS exclusion yielded 1077, 35, and 105 human discoveries at fixed 0.01, retaining astrocyte translation and oxidative phosphorylation evidence. At fixed 0.05, the astrocyte count fell from 39 to 15 after exclusion. Some translation elongation sets became ineligible. Estimated-correlation analyses remained nonsignificant. Figure 3 and Figure 4 distinguish these patterns; Figure 4 specifically shows sensitivity to RPL/RPS exclusion at a fixed intergene correlation of 0.01. Of 1071 primary astrocyte CAMERA-PR discoveries, 1016 retained their direction across available donor omissions, but only three remained significant in every variant. Figure 3 locates selected processes within the fixed-correlation evidence pattern.

### 2.4. Concordance Across Species Was Limited at the Gene Scale

Mapping retained 11,140 of 17,268 rat genes (64.5%) and 9739 of 13,237 mouse genes (73.6%), with 7437 common across primary species inputs. Human–mouse pairs contained 9404 genes. Moderated-*t* Spearman correlations were 0.068, −0.016, and −0.112 for astrocyte-, vascular-, and neuron-enriched fractions, with sign agreement 52.4%, 50.1%, and 46.3%. Human–rat correlations ranged from −0.105 to 0.099.

The strongest positive concordance paired STZ rat prefrontal cortex with diabetic mouse hippocampus (ρ = 0.242; sign agreement 58.8%; 15 jointly significant genes, 14 with a matching direction). GK striatum versus mouse hippocampus instead yielded ρ = −0.160 and 44.1% agreement. Figure 5 and Figure 6 summarize correlations and effect directions.

At a fixed correlation of 0.01, human astrocyte and mouse disease comparisons shared 66 significant pathways, all with matching competitive direction; signed evidence-score correlation was 0.424. This pathway agreement coexisted with the much weaker correlation across all shared genes in the same tissue comparison. Estimated-correlation tests yielded no jointly significant sets. Figure 5 shows gene-statistic correlations across disease comparisons. Figure 6 shows agreement in the direction of disease effects.

### 2.5. Fasting Opposed Selected Diabetes Effects Without Demonstrated Contraction

Diabetic hippocampus yielded 49 opposition genes under FDR adjustment within each criterion and 13 after joint adjustment across opposition and contraction. No primary contraction test was significant under either family. Table 1 gives the 13 joint-family candidates and their estimates.

Residual point estimates were closer to controls for 3400 genes (25.7%): 1947 without overshoot and 1453 after crossing the control value. Another 3845 overshot beyond the original distance, while 5992 moved in the direction of disease. These categories describe point estimates and do not establish significant contraction or restoration of expression.

*Abhd16a* had disease, IF, and residual coefficients of −0.282, 0.297, and 0.015, respectively, on the log_2_(FPKM + 1) scale. The point estimate crossed the control value but remained close to it. Opposition was significant (joint FDR of 0.012), whereas contraction was not (0.208); the residual-contrast FDR was 0.793. The fitted model implied a correlation between the sampling errors of the disease and IF contrasts of −0.512, compared with observed coefficient correlation −0.144.

Across 31 mouse omissions, joint-family opposition counts ranged from one to 50. *Abhd16a* remained significant in every variant; *Neurod6* and *Hnrnpa0* in 27. Up to two contraction discoveries appeared in two variants. One pathway, sperm flagellum assembly, showed opposing significant component directions under the initial fixed-correlation specification. Table 1 reports effects, intervals, and the conjunction FDR for the supported opposition genes. Appendix A Figure A2 shows their disease, IF and residual effects. Figure 7 relates the diabetic disease and IF coefficients to the point-estimate contraction region.

### 2.6. Dietary Expression Responses Were Heterogeneous Across Experiments

Ketogenic diet identified 999 genes among 14,895 tested, comprising 521 positive and 478 negative effects. Mouse omissions yielded 168–1903 discoveries, with coefficient correlations of 0.925–0.956 against the complete model. With BH adjustment within each contrast, APP/PS1 hippocampus yielded 74 disease genes, no IF genes, and 17 residual-comparison genes. The minimum IF FDR was 0.607. IF in wild-type and the disease-by-IF interaction yielded none; disease under IF yielded four. Table 2 summarizes the corrected primary gene and pathway results for both additional dietary experiments.

APP/PS1 mean aggregation yielded 72 disease genes; omission of FWT6, FAD3, or both yielded 98, 84, and 102. IF counts remained zero under BH adjustment within each contrast. The additional seven-contrast BH family (88,659 tests) yielded 180 genes responding to ketogenic diet. In APP/PS1, this family yielded 56 disease genes under ad libitum feeding, three IF genes, 29 residual-comparison genes, one IF gene in wild-type mice, two disease-by-IF interaction genes, and 11 disease genes under IF. The three APP/PS1 IF genes detected only with the combined family were *Kdm6b*, *Prkar2a* and *Myo16*. Their per-contrast BH FDR was 0.607, while combined-family FDR values were 0.04382–0.04882. Appendix A Table A2 gives their raw *p* values, ranks in both families and both adjusted values. These additional findings do not change the absence of IF discoveries under the reference per-contrast adjustment. Appendix A Figure A3 and Appendix A Table A3 report sensitivity results using BH adjustment within each contrast.

AD annotation correction resolved identical repetitions in 193 source rows, recovered five expressed genes, and removed 20 date-encoded labels, producing 12,294 features. Gene discovery counts and shared coefficients were unchanged across four tested annotation universes; Spearman correlations of moderated *t* statistics exceeded 0.99999999 in all comparisons. Joint opposition and contraction counts remained zero. One borderline IF pathway changed significance after correction (Appendix A Table A4).

### 2.7. Partial Dietary Concordance Did Not Extend Across Disease Contexts

Across 10,855 common genes, coefficients for diabetic IF and ketogenic diet had Pearson *r* = 0.205 and sign agreement 60.0%. The Spearman correlation of moderated *t* statistics was ρ = 0.284; 447 genes were significant in both experiments, including 359 with matching effect directions and 88 with opposite directions. The IF experiment sampled hippocampus, whereas the ketogenic-diet experiment sampled whole brain. This comparison therefore combines differences in dietary intervention, regional cell composition and experimental design; it does not isolate a shared effect of the two diets. IF responses in diabetic and APP/PS1 hippocampus were poorly concordant across 10,618 genes (*r* = −0.056, ρ = 0.015, sign agreement 45.8%), with no jointly significant genes. Their disease effects showed *r* = 0.085, ρ = 0.208, and sign agreement 56.4%, with nine jointly significant genes, six with matching effect directions.

At a fixed correlation of 0.01, CAMERA-PR identified 116 pathways for ketogenic diet, 28 for APP/PS1 disease, and three for IF in APP/PS1 mice; matched counts from CAMERA applied to expression matrices were identical. At fixed 0.05, CAMERA-PR retained 16 sets in the ketogenic experiment and no APP/PS1 sets. Estimated correlations yielded no discoveries (Appendix A Table A5).

Diabetic IF and ketogenic diet shared 59 significant pathways at a fixed intergene correlation of 0.01, all with matching directions, among 4214 common eligible sets. No shared pathway was supported under the estimated-correlation specification, so this overlap depends on the correlation treatment. Diabetic and APP/PS1 IF shared none among 4074 sets. Disease contrasts shared four, all with matching directions. Table 3 reports the concordance metrics and overlaps.

After RPL/RPS exclusion, the ketogenic experiment retained 87 discoveries with CAMERA-PR and 89 with CAMERA applied to expression matrices at fixed 0.01, including oxidative phosphorylation and respiratory electron transport. Their CAMERA-PR FDR values were 2.65 × 10^−7^ and 1.06 × 10^−4^ before exclusion, and 2.59 × 10^−6^ and 1.58 × 10^−4^ afterward; both retained upward competitive direction. No CAMERA-PR discoveries remained at 0.05. APP/PS1 contrasts retained none after exclusion at 0.01. Tested nonsignificant sets and sets that became ineligible were recorded separately. Neither dataset yielded discoveries with estimated correlations (Appendix A Table A6).

Corrected APP/PS1 IF discoveries had downward competitive direction: translation elongation, the EIF2AK4/GCN2 amino acid deficiency response, and a SARS-CoV-2 host-translation set (CAMERA-PR FDRs of 0.00887, 0.01193, and 0.04799). SRP targeting fell below significance after correction (FDR of 0.05088). These annotations did not survive fixed 0.05 or ribosomal exclusion. Selected reactive oxygen species, glutathione, and actin sets in the ketogenic experiment were nonsignificant at the initial specification. Table 3 gives exact gene concordance metrics and pathway overlaps across mouse experiments. Figure 8 displays the coefficient patterns across all shared genes underlying these comparisons. Figure 9 compares selected processes across the dietary and disease contexts under matched test specifications.

### 2.8. Response Geometry Across Disease Contexts and Exploratory Sex Modification

APP/PS1 disease and IF point estimates had opposite signs for 8700 genes (70.8%); 7039 (57.3%) had residual point estimates closer to wild-type ad libitum controls. Corresponding diabetic IF proportions were 54.7% and 25.7%. Nevertheless, no APP/PS1 gene met joint-family opposition or contraction FDR < 0.05 in any of the seven models, compared with 13 opposition genes and no primary contraction genes in diabetes. The fitted APP/PS1 model implied a correlation between the sampling errors of the disease and IF contrasts of −0.498. Figure 10 contrasts the descriptive and inferential results (Appendix A Table A7).

Formal APP/PS1 disease-by-sex testing identified 434 genes within that modifier family; six survived joint adjustment across all three modifiers: *Bmpr1b*, *Gli3*, *Gpr183*, *Lag3*, *Nr6a1*, and *Sox6*. Their male-minus-female coefficients ranged from −0.897 to −0.566, with a joint FDR of 0.03868–0.04589. No IF-by-sex modifier met the joint threshold (Appendix A Figure A4 and Appendix A Table A8 and Table A9).

## 3. Discussion

### 3.1. Dietary Convergence and Transfer Across Disease Contexts

IF and ketogenic diet shared part of their brain transcriptional response in diabetes, whereas IF responses showed poor agreement between the diabetic and APP/PS1 hippocampus. This distinction places the dietary response within its experimental disease context. A second finding concerns what that response means: 13 genes in diabetic mice showed opposing disease and IF effects, but neither primary fasting experiment established contraction toward controls.

Ketogenic diet provided a second dietary experiment in diabetes, with modest overall agreement with IF and 359 jointly significant genes with matching directions. The two studies sampled whole brain and hippocampus, respectively, and differed in dietary protocol and experimental design. The observed agreement therefore identifies a shared component across experiments but cannot separate dietary effects from regional cell composition and other study differences. In the ketogenic-diet omission analyses, effect coefficients were more stable than discovery counts, indicating that effect estimation and significance thresholds capture different aspects of reproducibility.

The 59 pathways with matching enrichment directions were identified at a fixed intergene correlation of 0.01 and were not supported when correlations were estimated from the data. Their interpretation is conditional on that specification, and overlapping pathway annotations can contain many of the same genes. The gene-level agreement, together with the 88 jointly significant genes showing opposite directions, supports partial agreement with substantial differences between the experiments. These results identify candidates for direct comparison of IF and ketogenic diet in matched tissues and experimental conditions.

IF and ketogenic feeding can both alter fuel availability. Fasting on alternate days supported neuronal resistance to injury caused by kainic acid, and cyclic ketogenic feeding improved memory in aging mice [10,11]. The present results define its transcriptomic extent.

The APP/PS1 experiment tests how far this convergence extends. IF coefficients agreed poorly with those in the diabetic hippocampus, and no significant IF pathways overlapped at a fixed correlation of 0.01. In these experiments, IF effects showed little agreement across the two disease contexts. This result does not establish that IF is ineffective in AD or that dietary responses cannot be shared under other experimental conditions.

Disease effects also showed weak agreement between *db*/*db* and APP/PS1 hippocampus. The two fasting experiments began from different expression patterns, which may contribute to the differences in their dietary responses. Both comparisons favor interpreting IF within the disease context in which it was applied. The APP/PS1 experiment tests whether the diabetic findings extend to an AD model. The current data cannot attribute the differences to the genotype alone because the cohorts and feeding schedules also differ. Differences in statistical power also constrain comparisons based on significant-gene overlap. The absence of shared discoveries does not establish equivalence to no IF response in APP/PS1 mice.

Dietary timing can nevertheless affect AD phenotypes under other conditions—feeding restricted to the active phase altered hippocampal transcription in APP23 mice and reduced amyloid burden and improved memory in APP knock-in mice [12]. Differences in model and schedule may contribute to the present contrast. Expression contraction and behavioral or pathological improvement are distinct endpoints.

Impaired cerebral insulin and IGF-1 signaling provides a rationale for comparing diabetes and AD [8,9]. Reduced ex vivo signaling responses were demonstrated in postmortem hippocampal formation and cerebellar cortex from AD cases without diabetes [9]. This supports the investigation of shared metabolic features. The current expression comparisons test transferability.

### 3.2. Covariance and Gene Membership Shape Pathway Evidence

Pathway evidence was sensitive to intergene correlation across all five datasets, including the ketogenic experiment analyzed from raw counts. Many discoveries obtained with fixed correlations did not remain significant at a higher fixed value or with estimated residual correlations. Apparent pathway agreement can coexist with weak agreement across all shared genes.

Estimated correlations provide an alternative specification. Their precision depends on replication, and these tests also use a finite number of residual degrees of freedom. Matched analyses using expression matrices indicate that the large change in discoveries is not explained solely by exchanging gene-statistic ranks for expression inputs. Fixed and estimated results together define the conditions under which a pathway association is supported.

Removing canonical RPL/RPS genes further distinguished the dietary responses. Respiratory pathways remained significant in the ketogenic experiment at a fixed correlation of 0.01, whereas the APP/PS1 discoveries did not. The former association, therefore, extended beyond the excluded ribosomal genes under that test specification. Removing genes also changes set eligibility and the competitive background. The translation annotations, including the SARS-CoV-2 host-translation label, should accordingly be interpreted in light of their constituent genes.

Oxidative phosphorylation and respiratory electron transport retained their upward competitive ranks after exclusion of RPL/RPS in the ketogenic experiment. That indicates that the expression of respiratory machinery is a specific component of the dietary response, describing the relative ranks of respiratory gene statistics within the measured brain transcriptome. Selected reactive oxygen species and glutathione sets were nonsignificant—the current evidence is more specific to respiratory gene expression than to a general antioxidant response.

Annotations concerning translation in APP/PS1 mice showed a different response to IF. Their initial competitive direction was downward, but the evidence did not remain significant after excluding ribosomal genes. This suggests that the observed enrichment depends substantially on the representation of ribosomal genes. The contrasting results of the exclusion analysis indicate that the respiratory response to ketogenic feeding was more robust under the tested specification than the translation response to IF in APP/PS1 mice.

### 3.3. Contribution

The source studies characterized brain expression and dietary outcomes within their own experimental settings [1,2,3,6,7]—earlier secondary analyses examined shared disease signatures or immune candidates [4,5]. The present study connects three biological observations: limited agreement in expression related to disease across species, partial agreement between IF and ketogenic diet in diabetes, and little agreement in IF effects between diabetic and APP/PS1 hippocampus. This combined assessment identifies candidate genes and processes for direct comparative studies while defining limits to their transferability. A substantial dietary response was not sufficient to establish a reduction in the original expression difference from controls.

Several features may contribute to the differences between human and rodent findings. The human cohort comprised older postmortem donors and enriched temporal cortical fractions, whereas the rodent datasets used distinct disease models and brain sampling schemes. Diabetes duration, treatment and comorbid conditions may add heterogeneity in humans. The small donor cohort also limits precision after covariate adjustment. These factors could reduce agreement or statistical power, and the available data cannot separate their contributions from biological differences between models. The absence of significant human gene discoveries should therefore be interpreted within this sampling and analytical setting.

The opposition–contraction distinction is central to this contribution. A large IF response and opposite disease–IF signs did not establish a smaller residual distance. *Abhd16a* illustrates the difference: its residual estimate was near zero, and opposition was stable across all mouse omissions, but contraction remained nonsignificant. A nonsignificant residual test also does not establish equivalence, which requires a specified margin and an appropriate test [13].

The two fasting experiments illustrate why these distinctions matter. Diabetic mice showed an extensive IF response, including a smaller subset of genes with significant opposition between disease and IF effects. APP/PS1 mice had a larger proportion of point estimates closer to controls, yet no significant opposition or contraction. A comparison based only on those proportions would thus give a different impression from the inferential results—reporting response magnitude, direction, and distance from controls separately makes the dietary findings interpretable and supplies a defined endpoint for subsequent intervention studies.

Contraction is a defined expression endpoint; functional benefit can occur without it, and contraction alone would not establish functional rescue.

The factorial APP/PS1 design also separated disease modification from treatment modification. Six exploratory disease-by-sex modifiers survived joint testing across the three sex modifiers; no IF-by-sex modifier met that threshold. The disease-by-IF interaction yielded no gene discoveries with BH adjustment within that contrast, although two genes met the additional seven-contrast BH threshold. The disease-by-sex candidates identify heterogeneity in disease effects and warrant replication; sex-stratum discovery counts should not be interpreted as differential fasting benefit.

### 3.4. Strengths and Limitations

The study connects five datasets through common endpoints while preserving their sampling units, measured universes, and source testing families, and comparisons across all shared genes avoid selection on overlapping significant lists. Matched pathway specifications and annotation correction assess robustness, and conjunction tests retain contrast covariance. Together, these features provide an integrated assessment of dietary association, transferability, and contraction.

Biological heterogeneity limits a simple attribution of cross-study differences. Species, genetic background, disease model, age, tissue composition and feeding schedule vary together. In particular, the IF–ketogenic comparison pairs hippocampus with whole brain, so agreement cannot be attributed uniquely to the dietary intervention. The profiles represent bulk tissues or enriched cell populations and do not resolve individual cells or cell states. Regional cell composition and incomplete cellular enrichment may mask cell-specific responses. Human clinical and postmortem covariates could be addressed only to the extent permitted by the small cohort and available metadata. The ketogenic experiment has four animals per group and no healthy comparator; APP/PS1 and diabetic IF cohorts are separate, but their source studies share investigators. Deposited processed arrays and FPKM restrict reconstruction of sequencing precision and source preprocessing. Mapping uniqueness is specific to the frozen annotation cache, and correction did not resolve missing or distinct-symbol AD assignments. In GSE125387, ribosomal exclusion removed only *Rpl6l*, so this sensitivity does not establish independence from cytosolic ribosomal genes. The origin of the GSE125387 figure-caption count discrepancy remains unresolved despite agreement between the sample tables deposited and associated with the publication. Comparable tissues, balanced disease-by-intervention designs, and direct metabolic and functional measurements would test the biological interpretation of the identified expression patterns.

## 4. Methods and Materials

### 4.1. Datasets and Biological Sampling Units

Five datasets were obtained from the Gene Expression Omnibus (GEO) [14,15]: GSE161355, GSE34451, GSE125387, GSE249467, and GSE164461. The study used deposited expression and metadata without new participant or animal experiments. Analyses were exploratory and not preregistered. Source publications describe tissue collection and experimental procedures [1,2,3,6,7].

GSE161355 contained astrocyte-enriched, vascular-enriched, and neuron-enriched temporal cortical fractions from six donors with diabetes and five controls, totaling 33 arrays. Each fraction was modeled separately, with donors as biological units. Deposited case codes were linked to the source demographic table. Age and sex were included without imputation; postmortem interval and tissue pH were recorded but not included in the primary model. Although the abstract described six controls, the microarray methods specified six donors with T2D and five controls [2], consistent with the deposited cohort analyzed here. Available donor-level demographic and postmortem characteristics are reported in Appendix A Table A10. The available metadata did not permit systematic adjustment for diabetes duration, complications, treatment, occupational exposure or environmental factors.

GSE34451 included hippocampus, prefrontal cortex, and striatum from control Wistar rats, Wistar rats treated with streptozotocin, and Goto–Kakizaki rats. Each regional group comprised three independently measured pools of tissue from three animals each. Pools were the statistical units. Regions were modeled separately; pairing across regions was not established. GSE125387 contained hippocampal expression from 11 db/m controls, 10 db/db mice, and 10 db/db mice subjected to IF for 28 days [3]. Sample assignments were checked against the GEO series and individual sample records, the deposited FPKM workbook, and Supplementary Data S1 and S2 accompanying the source publication. These sources consistently identified the same 31 samples and groups. All sample labels, gene identifiers and expression values in the published FPKM table matched the deposited workbook. The source methods describe 10–11 mice per group for the multiomics cohort, whereas the caption of Figure 3a in the source publication [3] reports 11, 9, and 11. No documentation for individual samples supported an alternative assignment. A numerical audit confirmed the imported values and the sample assignments in the saved model. The deposited assignments were retained; their mapping to GSM accessions and expression columns is provided in Appendix A Table A11.

GSE249467 contained whole-brain raw RNA-sequencing counts from eight male db/db mice, four receiving standard diet and four ketogenic diet. Feeding began at nine weeks of age and continued to six months [6]. The contrast compared ketogenic with standard diet within diabetes.

GSE164461 contained hippocampal expression from 55 mice: 15 wild-type ad libitum, 13 wild-type IF, 14 APP/PS1 ad libitum, and 13 APP/PS1 IF, comprising 27 females and 28 males. AD contrast labels denote the APP/PS1 genotype. The intervention lasted 16 weeks beginning at four months of age [7]. Samples were separate from GSE125387.

### 4.2. Expression Preprocessing and Gene Representation

Human expression was deposited after robust multiarray averaging and was not log-transformed again. Rat intensities were transformed to log_2_ and quantile-normalized within each region; log_2_-only inputs were retained for sensitivity analysis. Microarray probes with unambiguous assignments were combined by averaging log-scale expression. Sensitivity models selected the highest-mean probe independently of group labels, reusing that selection across rat normalization variants.

GSE125387 FPKM values were transformed as log_2_(FPKM + 1). Genes required FPKM ≥ 1 in at least 10 samples. Ambiguous symbols were excluded; repeated unambiguous symbols were represented by the highest mean transformed expression across all samples. Opaque source identifiers were not treated as Ensembl or Entrez identifiers. Primary universes contained 20,848 genes per human fraction, 17,268 per rat region, and 13,237 in GSE125387. Raw counts were not used for this dataset. Coefficients for FPKM inputs were differences in mean log_2_(FPKM + 1).

GSE249467 counts underwent edgeR expression filtering [16,17], trimmed mean of M-values normalization [18], and voom weighting [19], retaining 14,895 of 17,983 rows. GSE164461 used log_2_(FPKM + 1) with FPKM ≥ 1 in at least 13 samples. The highest-mean row represented duplicate unambiguous symbols; mean log-scale aggregation provided a sensitivity representation. The corrected 12,294-feature universe was fixed across sample-omission and sex-stratified models.

The AD annotation audit resolved identical repeated symbols and removed labels stored as dates, identified from spreadsheet cell data types. Automatic conversion of gene symbols to dates is a documented spreadsheet error [20]. Four universes were compared: original (12,309), five recovered genes added (12,314), 20 date labels removed (12,289), and combined correction (12,294). Baseline refitting checked agreement with saved analyses. Date labels were removed without reassignment. Distinct-symbol assignments were not copied to multiple genes, and missing MSTRG identities were not mapped from numeric suffixes. Corrected models were propagated to pathway, concordance, sex-modifier, and opposition–contraction analyses.

### 4.3. Differential Expression and Sensitivity Models

Expression was modeled with limma empirical Bayes moderation and robust variance estimation [21,22]. Robust moderation was applied to gene variance estimation, not to sample regression. Unweighted matrices used variance trends that depended on abundance; voom-weighted GSE249467 models used no additional trend. The primary human design included group, sex, and centered age (seven residual degrees of freedom), with simpler covariate designs retained as sensitivities. Rat and GSE125387 designs included group only. Rat contrasts compared each disease model with Wistar controls. GSE125387 contrasts compared db/db with db/m, db/db mice subjected to IF with untreated db/db mice, and db/db mice subjected to IF with db/m.

Primary models retained all samples. Sensitivities used donor or unit omission, alternative covariates and gene representations, and omission of two flagged astrocyte controls. Gene significance used Benjamini–Hochberg (BH) adjustment across the complete tested universe within each contrast at an FDR < 0.05 [23], without an additional fold-change threshold. Confidence intervals were pointwise 95% moderated intervals.

For the human source-criteria audit, all 54,675 deposited probe sets were tested without symbol filtering or gene aggregation. Unadjusted Student *t* tests and the original 1.2-fold threshold were applied, followed by a separate BH adjustment over all probe sets within each fraction. Raw CEL preprocessing was not repeated.

GSE249467 used a group-only design (six residual degrees of freedom) and eight leave-one-mouse-out fits. GSE164461 used group plus sex (50 residual degrees of freedom). Six biological contrasts estimated disease under ad libitum feeding, IF within APP/PS1, APP/PS1 IF versus wild-type ad libitum, IF within wild-type, disease-by-IF interaction, and disease under IF. The auxiliary contrast 2*D* + *I* entered the contraction test. Sensitivities used mean aggregation, sex strata, and omission of samples FWT6 and FAD3 flagged during quality control individually and together. Gene discoveries were defined using BH adjustment within each contrast. An additional BH family combined all 88,659 primary gene tests across the seven biological contrasts from these two datasets; these results were reported separately. BH-adjusted values depend on the complete ordered *p*-value distribution within the selected testing family. Adding contrasts can introduce many small *p* values and change their ranks sufficiently to produce smaller adjusted values despite the larger number of tests. The combined family controls the FDR across the pooled gene-contrast tests; it does not provide separate FDR control for the IF contrast. Per-contrast results remained the reference for contrast-specific gene discoveries.

Exploratory sex-modifier models used eight genotype-by-diet-by-sex group means (47 residual degrees of freedom). Contrasts estimated male-minus-female differences in disease effects and IF effects within each genotype. BH adjustment was applied within each modifier and jointly across all three modifiers (36,882 tests).

### 4.4. Competitive Gene Set Analysis

Gene sets were obtained from the Molecular Signatures Database (MSigDB) [24] through msigdbr 26.1.1 [25], retaining Hallmark [26], Reactome [27,28], and GO biological process [29,30] collections. Membership was fixed to human MSigDB release 2026.1.Hs. Rodent sets used its supplied species symbols and ortholog annotations. Eligible sets contained 15–500 measured genes and at least 10% of their source membership.

CAMERA-PR tested complete moderated *t*-statistic vectors at fixed intergene correlations of 0.01 and 0.05. Rank CAMERA applied to expression matrices used fixed and residual correlations estimated separately for each gene set [31]. The three diabetes datasets used both fixed values and estimated correlation; the two additional dietary datasets used fixed 0.01 and estimated correlation. Unweighted matrices used a variance trend; GSE249467 retained voom weights without an additional trend. Negative correlation estimates were bounded at zero for variance inflation, with raw estimates saved. Estimated-correlation tests used finite residual degrees of freedom.

BH adjustment covered all eligible sets from the retained collections within each model and contrast. Additional families combined 57,594 primary tests using ranks from the three diabetes datasets and 29,811 from the additional dietary datasets. Per-contrast and combined-family results were reported separately. Integration retained source FDR values without recalculation on selected or overlapping sets. Verified rank continuity-correction *p* values above 1 were bounded to 1 and recorded. Competitive direction described relative gene-statistic ranks; selected-process displays retained this direction and the corresponding source FDR.

### 4.5. Sensitivity to Cytosolic Ribosomal Genes

Canonical cytosolic RPL/RPS genes were excluded using a strict symbol pattern, with uppercase classification and native-symbol matching. RPS6 kinases, mitochondrial MRPL/MRPS genes, and most ribosomal pseudogenes were retained. Exclusion removed 66 genes per human fraction, 80 per rat region, and one gene, *Rpl6l*, from GSE125387. Membership, eligibility, background, and the FDR were recomputed; ineligible sets were recorded as unavailable.

Both additional dietary datasets were tested before and after RPL/RPS exclusion with CAMERA-PR and CAMERA applied to expression matrices. GSE249467 retained the original voom weights; moderation was recomputed on retained rows. Appendix A Table A12 reports universe and eligibility counts.

### 4.6. Cross-Species Integration

Human and rodent symbols were mapped through the frozen MSigDB annotation cache, retaining mappings unique in both directions within that cache. Missing and ambiguous mappings were recorded separately. Aliases and case conversion were not used to force matches.

Cross-species integration retained source coefficients, moderated *t* statistics, and FDR values. Descriptive metrics comprised Spearman correlations of moderated *t* statistics, effect-sign agreement, agreement among the 500 strongest statistics in each pair, and genes significant in both source comparisons. The integration included 51 comparisons, 27 using primary inputs. Pathway concordance retained matched test specifications, source FDR, and competitive direction.

Mouse comparisons matched unambiguous symbols across complete universes and reported coefficient Pearson correlations, Spearman correlations of moderated *t* statistics, sign agreement, and jointly significant genes. Comparisons paired diabetic IF with ketogenic diet, diabetic IF with APP/PS1 IF, and the two disease effects. The FDR was not recalculated on mapped subsets. No pooled effects or correlation significance tests assuming independent genes were calculated.

### 4.7. Opposition and Contraction After Intermittent Fasting

In GSE125387, *D* was db/db minus db/m, *I* was db/db mice subjected to IF minus untreated db/db mice, and *R* was db/db mice subjected to IF minus db/m. In GSE164461, *D* was APP/PS1 ad libitum minus wild-type ad libitum, *I* was APP/PS1 IF minus APP/PS1 ad libitum, and *R* was APP/PS1 IF minus wild-type ad libitum. In both experiments, *R* = *D* + *I*. Opposition required *D* > 0 and *I* < 0, or *D* < 0 and *I* > 0. Contraction required |*R*| < |*D*|. For nonzero *D*, this requires opposition and *F* = 2*D* + *I* to retain the sign of *D*: *D* > 0, *I* < 0, *F* > 0, or *D* < 0, *I* > 0, *F* < 0.

Each branch used the maximum constituent one-sided moderated *p* value; branches were combined with a Bonferroni factor of two. *F* variance used the fitted contrast covariance, posterior residual variance, and moderated degrees of freedom. Tests covered all genes. BH adjustment was applied within each criterion and jointly across opposition and contraction, comprising 26,474 tests in GSE125387 and 24,588 in corrected GSE164461. Joint-family results defined primary candidates.

The posterior variance and contrast covariance were taken from each fitted model. Disease, IF, and residual effects used the same fitted samples and gene universe. In the APP/PS1 analysis, the geometry used wild-type ad libitum as the control reference; wild-type IF entered separate dietary and interaction contrasts. Omission sensitivities refitted the models before repeating the conjunction calculations.

Descriptive categories separated movement toward controls without overshoot, overshoot remaining closer, overshoot beyond the original distance, and movement in the disease direction. Tests were repeated in 31 GSE125387 omission models and seven GSE164461 primary and sensitivity models. No equivalence margin or pathway contraction test was specified.

### 4.8. Computational Reproducibility

Configuration seed was 20261011. Deterministic calculations used R 4.6.1, limma 3.68.5, msigdbr 26.1.1, and statmod 1.5.2. Cached annotations, input hashes, sessions, and integrity checks covered model summaries, testing families, annotation coverage, and table consistency. Deposition details appear in the availability statement.

### 4.9. Analytical Hierarchy

All analyses were exploratory and not preregistered. The following hierarchy distinguishes the reference analyses from sensitivity analyses and additional inference; it does not imply prospective prespecification.

Reference analyses used the complete cohorts, the stated gene representations and covariate designs, and BH FDR < 0.05 within each gene contrast. Primary opposition and contraction candidates required BH FDR < 0.05 jointly across both criteria within each fasting dataset. Cross-experiment concordance was descriptive and retained the source gene and pathway FDR values.

Sensitivity analyses examined sample or donor omission, alternative covariates, normalization, gene representation, annotation correction, RPL/RPS exclusion and alternative intergene-correlation specifications. Pathway discoveries were interpreted separately for each specification. Significance at a fixed correlation of 0.01 was not treated as evidence of robustness to estimated correlations.

Additional analyses comprised the pooled gene and pathway testing families, exploratory sex modifiers and selected-process displays. The pooled gene family did not replace per-contrast adjustment. Descriptive point-estimate proximity to controls was distinguished from significant contraction.

## 5. Conclusions

Across the analyzed experiments, IF and ketogenic diet showed limited gene-level agreement in diabetes. Their shared pathway findings depended on the intergene-correlation specification. IF effects showed little agreement between diabetic and APP/PS1 hippocampus. Thirteen diabetic genes showed significant opposition of disease and IF effects, but neither primary fasting experiment established contraction toward controls. These findings do not establish transcriptomic restoration or exclude functional dietary benefits. Differences in tissues, models and feeding protocols limit attribution of the observed agreement or disagreement to a single biological factor.

## Figures and Tables

**Figure 1:**
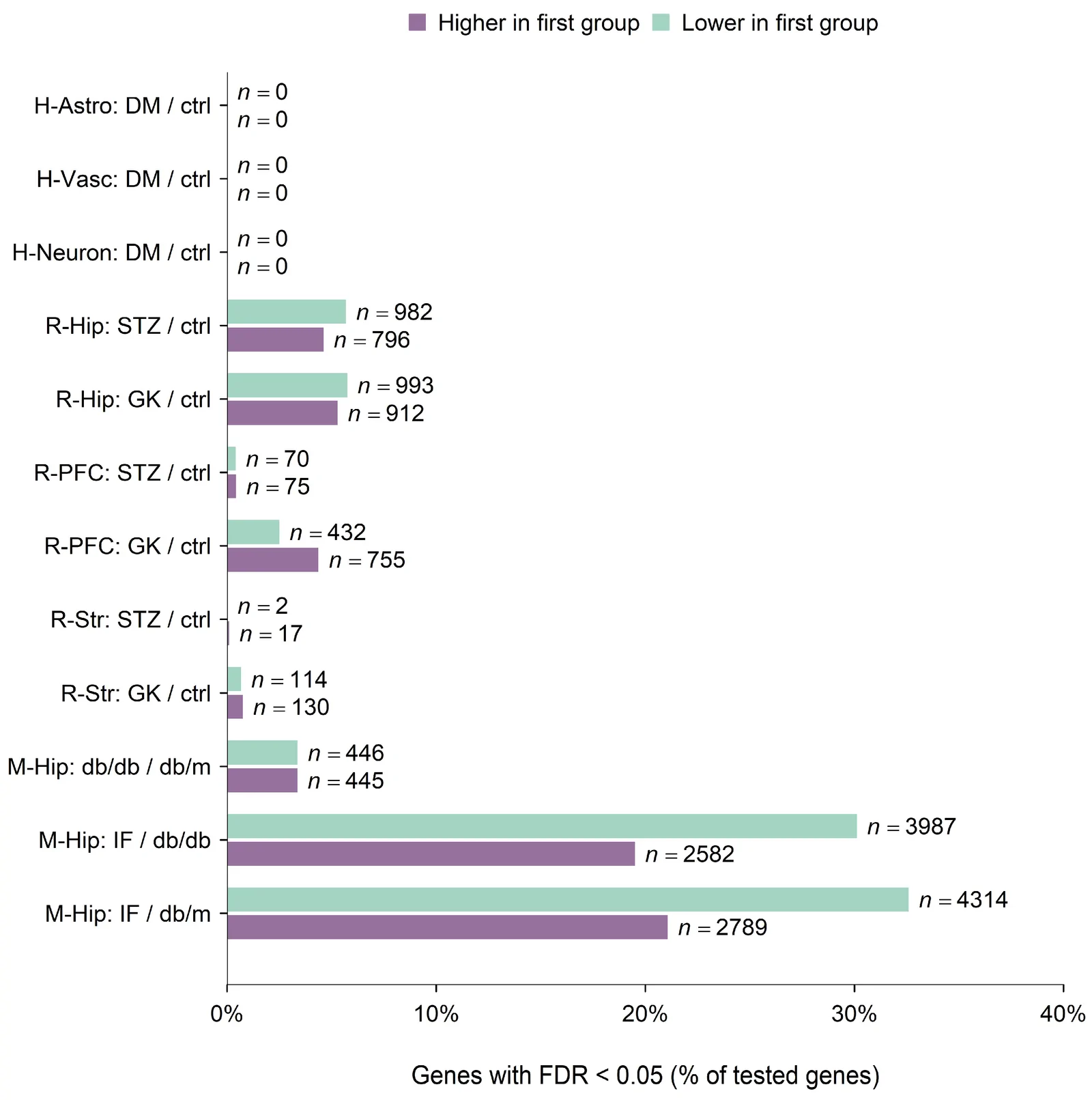
Gene expression differences in the primary models. Bar length is the percentage of all tested genes with per-contrast FDR < 0.05 in the indicated direction; *n* is the corresponding gene count. Purple indicates higher and green lower expression in the first group relative to the second. A slash denotes a contrast, not an expression ratio. H-Astro, H-Vasc, and H-Neuron denote human astrocyte-, vascular-, and neuron-enriched fractions; R-Hip, R-PFC, and R-Str denote rat hippocampus, prefrontal cortex, and striatum; M-Hip denotes mouse hippocampus. DM, diabetes; STZ, streptozotocin; GK, Goto–Kakizaki; IF, intermittent fasting; ctrl, control. db/db, diabetic mice; db/m, nondiabetic controls; FDR, false discovery rate, adjusted by the Benjamini–Hochberg procedure. Higher and lower counts are separate, noncumulative percentages. Rat units were pools, and the human fractions shared donors.

**Figure 2:**
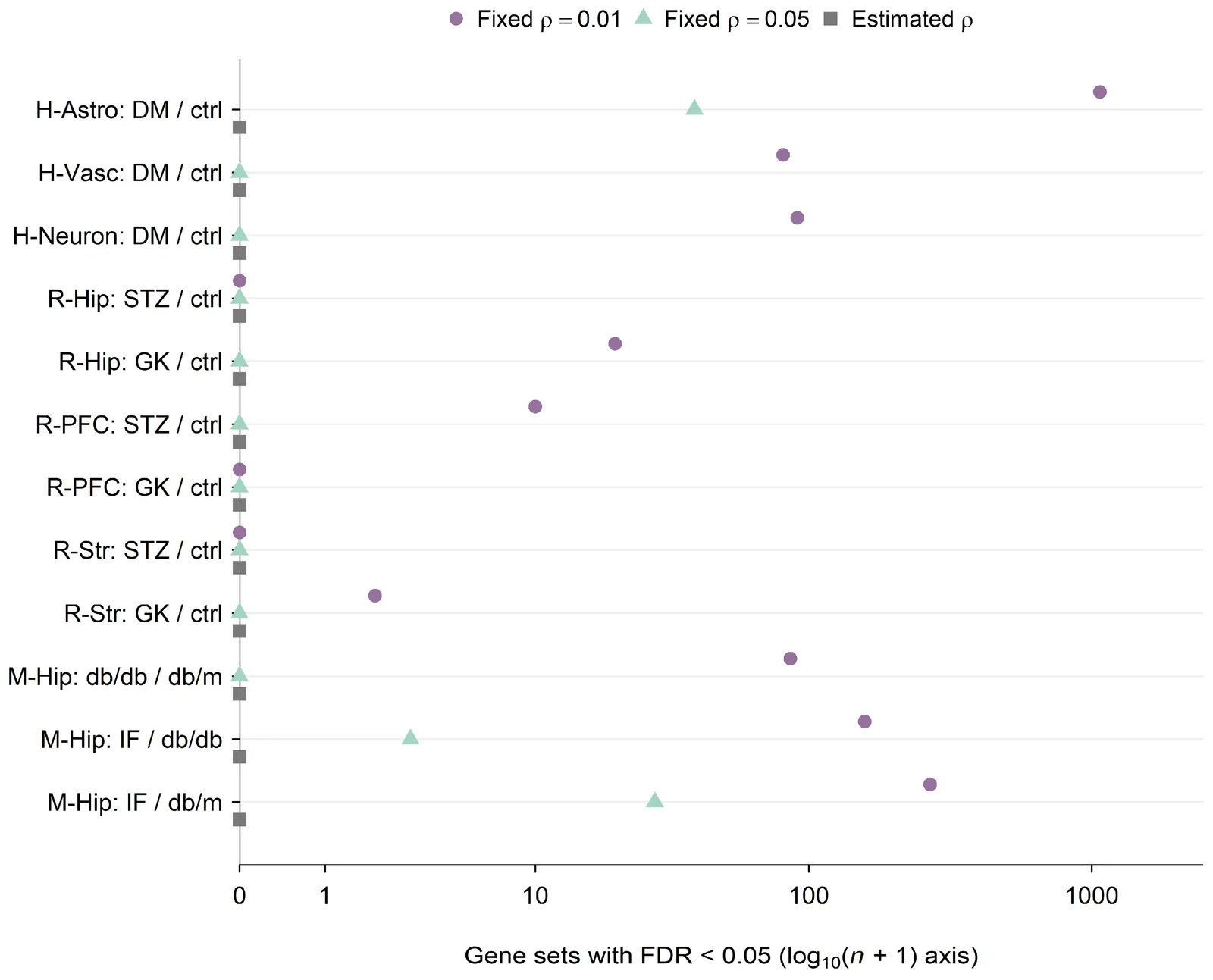
Sensitivity of pathway discovery counts to correlation treatment in rank CAMERA applied to expression matrices. Purple circles denote fixed intergene correlation ρ = 0.01, green triangles fixed ρ = 0.05, and gray squares residual correlations estimated separately for each gene set. Vertical offsets separate settings without changing counts. The horizontal axis is log_10_(*n* + 1), where *n* is the gene-set count and ticks display counts. Benjamini–Hochberg adjustment covers all eligible collections within each comparison. H-Astro, H-Vasc, H-Neuron: human astrocyte-, vascular-, and neuron-enriched fractions; R-Hip, R-PFC, R-Str: rat hippocampus, prefrontal cortex, and striatum; M-Hip, mouse hippocampus; STZ, streptozotocin; GK, Goto-Kakizaki; DM, diabetes; IF, intermittent fasting; ctrl, control; db/db, diabetic mice; db/m, nondiabetic controls. IF labels denote db/db mice subjected to IF. A slash denotes a comparison of the first group with the second; it does not denote an expression ratio. CAMERA, correlation-adjusted mean rank gene set test; FDR, false discovery rate.

**Figure 3:**
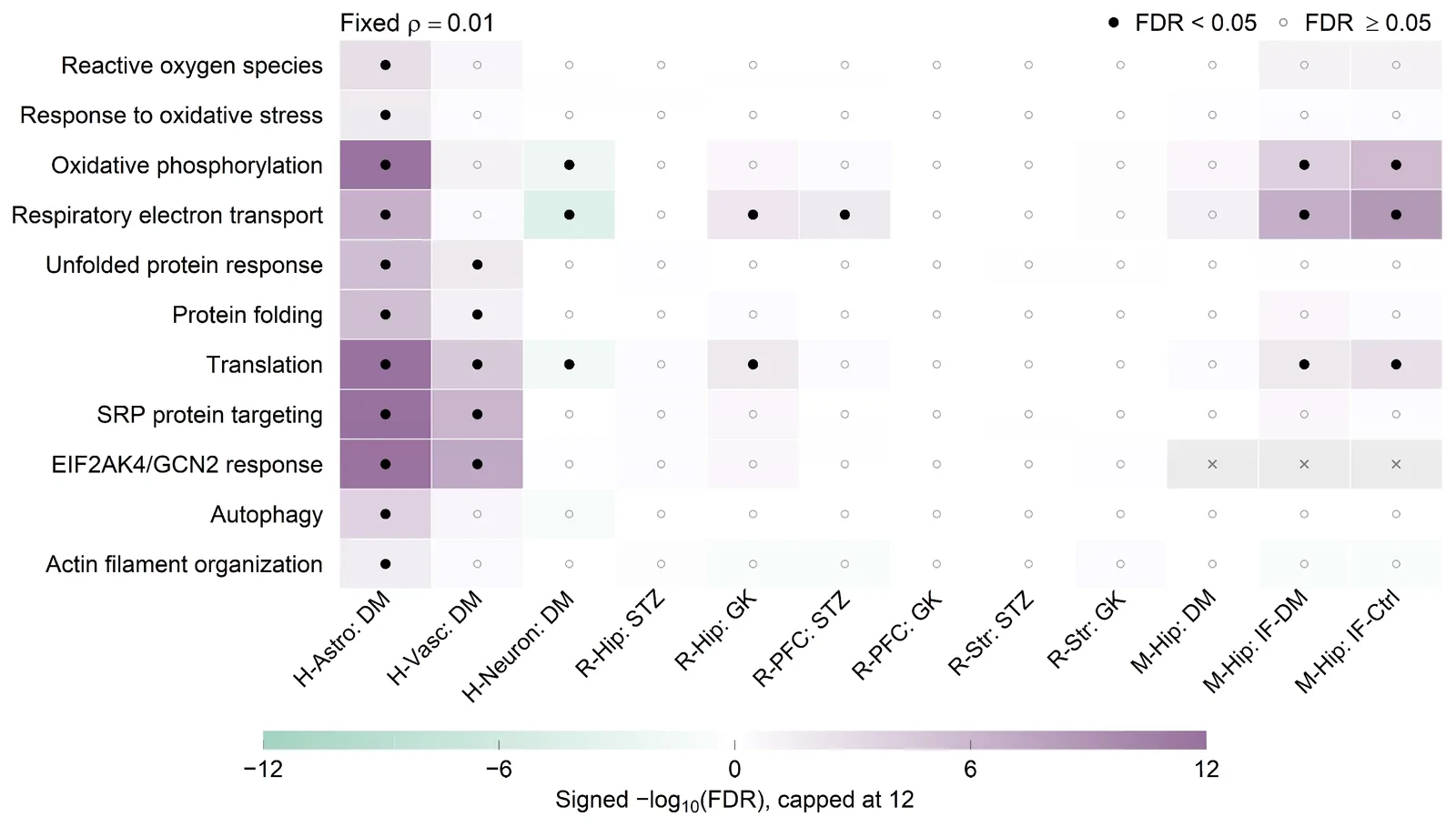
Selected processes at fixed intergene correlation ρ = 0.01 in rank CAMERA applied to expression matrices. Color shows competitive direction multiplied by −log_10_(FDR), capped at magnitude 12: purple denotes higher and green lower ranks in the first comparison group. Filled circles mark FDR < 0.05, open circles mark tested results with FDR >= 0.05, and gray crosses mark sets not tested. Scores do not measure pathway activity. The 11 processes were selected for exploratory presentation. H-Astro, H-Vasc, H-Neuron: human astrocyte-, vascular-, and neuron-enriched fractions; R-Hip, R-PFC, R-Str: rat hippocampus, prefrontal cortex, and striatum; M-Hip, mouse hippocampus; STZ, streptozotocin; GK, Goto–Kakizaki; IF, intermittent fasting; FDR, false discovery rate. DM denotes the disease-versus-control contrast, IF-DM denotes diabetic db/db mice subjected to IF versus untreated db/db mice, and IF-Ctrl denotes db/db mice subjected to IF versus nondiabetic db/m mice. SRP, signal recognition particle; EIF2AK4/GCN2, eukaryotic translation initiation factor 2 alpha kinase 4/general control nonderepressible 2. White or near-white cells indicate weak signed evidence, not necessarily an absent effect.

**Figure 4:**
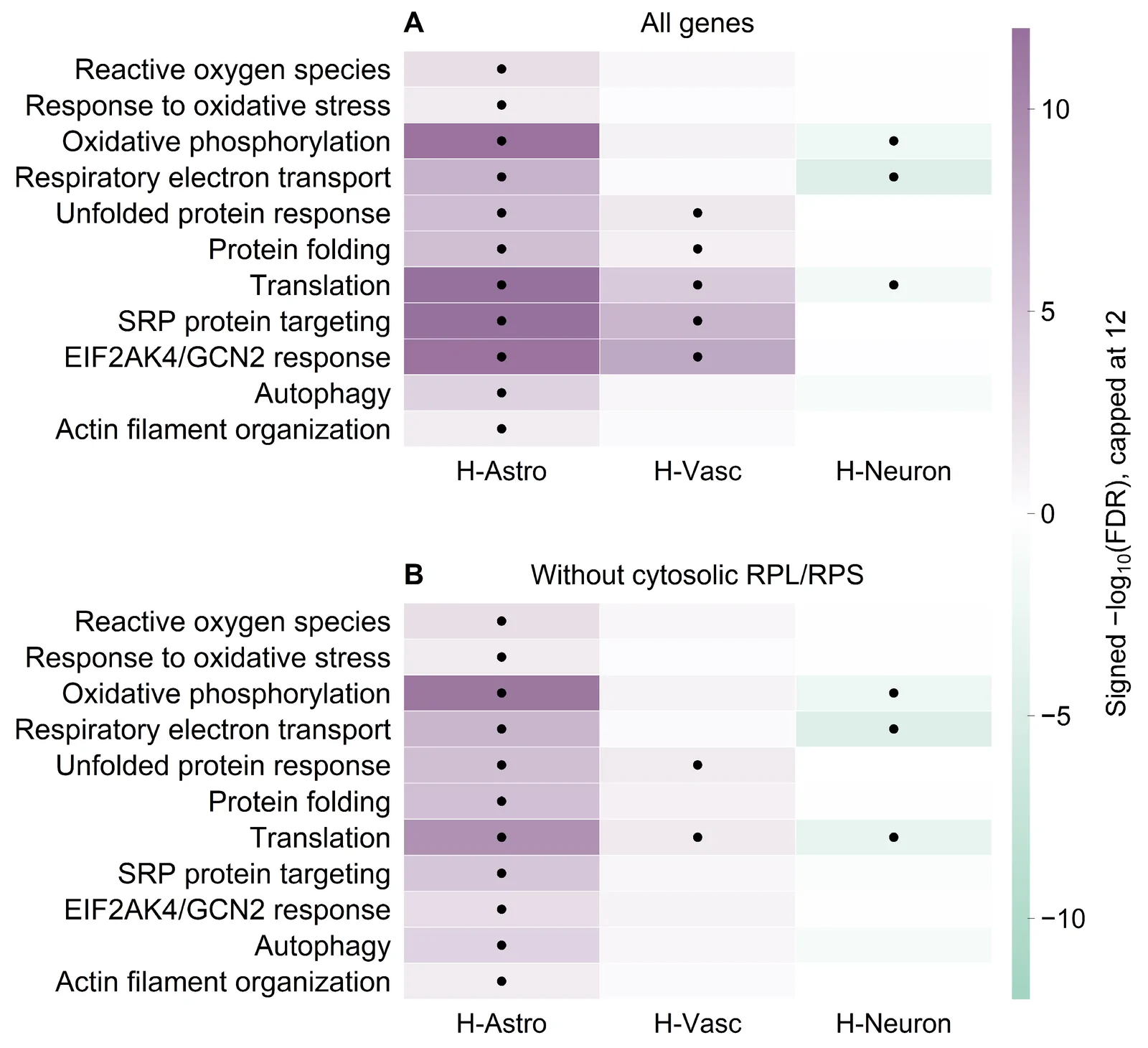
Sensitivity of selected human processes to cytosolic RPL/RPS exclusion. Panels (**A**,**B**) use all genes and the reduced universe, respectively, in rank CAMERA applied to expression matrices at a fixed correlation of 0.01. Purple denotes higher and green lower competitive ranks; black circles mark FDR < 0.05 and gray crosses ineligible sets. Color is signed −log_10_(FDR), capped at magnitude 12. H-Astro, H-Vasc, and H-Neuron denote astrocyte-, vascular-, and neuron-enriched fractions; SRP, signal recognition particle; EIF2AK4/GCN2, eukaryotic translation initiation factor 2 alpha kinase 4/general control nonderepressible 2; RPL/RPS, cytosolic ribosomal protein large/small subunits. Unmarked cells are tested nonsignificant results. Comparisons are diabetes minus control within each fraction; FDR, false discovery rate. This analysis assesses sensitivity to RPL/RPS exclusion at the stated fixed correlation; it does not establish robustness to estimated correlations.

**Figure 5:**
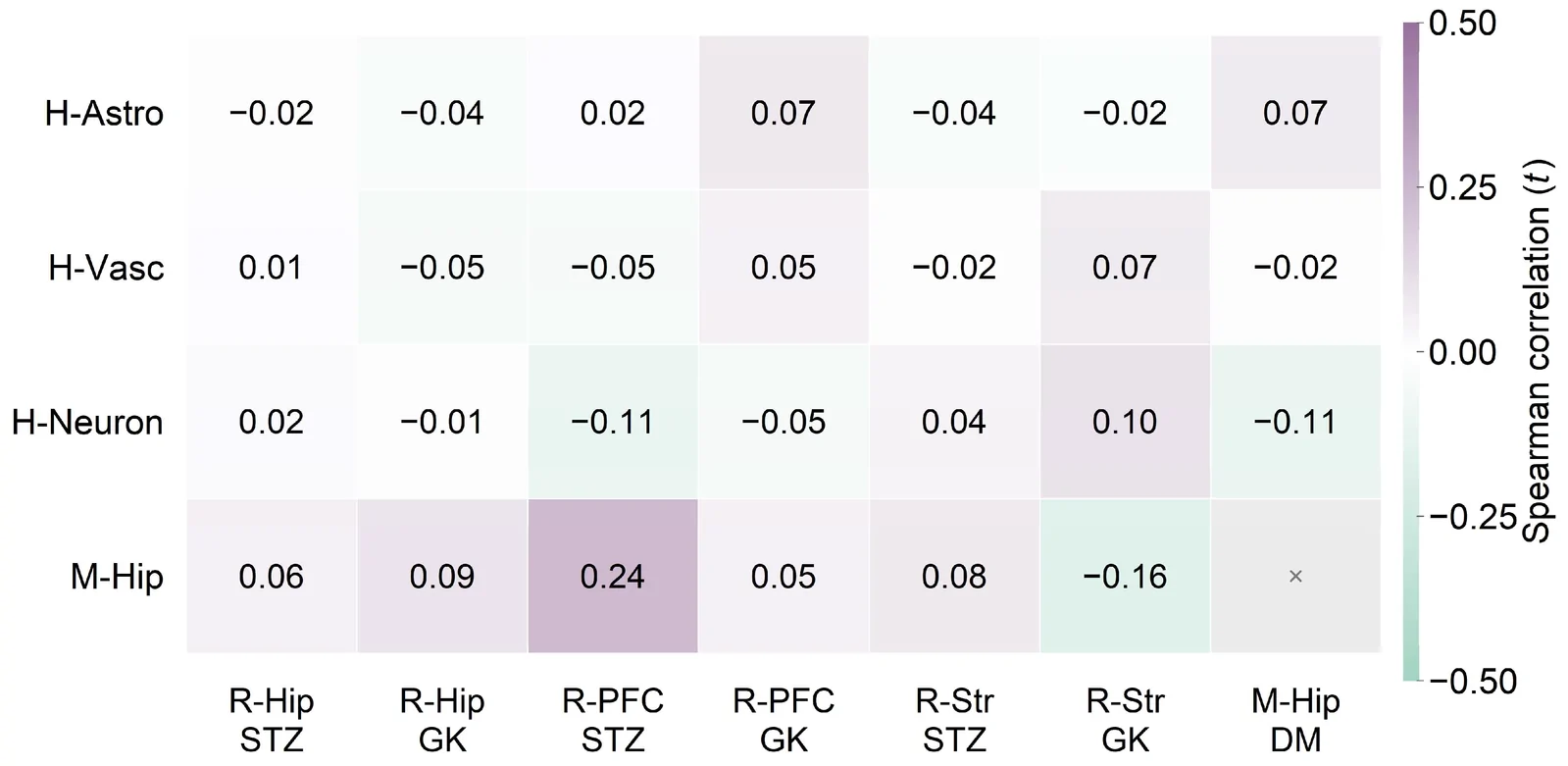
Cross-species concordance of primary disease contrasts. Cells show Spearman correlations of moderated *t* statistics for common uniquely mapped genes. Purple denotes more positive and green more negative correlations, with white at zero and color limits −0.5 to 0.5. Printed numbers give correlations; a gray cross marks the unperformed mouse self-comparison. H-Astro, H-Vasc, H-Neuron: human astrocyte-, vascular-, and neuron-enriched fractions; R-Hip, R-PFC, R-Str: rat hippocampus, prefrontal cortex, and striatum; M-Hip, mouse hippocampus; STZ, streptozotocin; GK, Goto–Kakizaki; DM, diabetes. Mappings used the frozen MSigDB cache.

**Figure 6:**
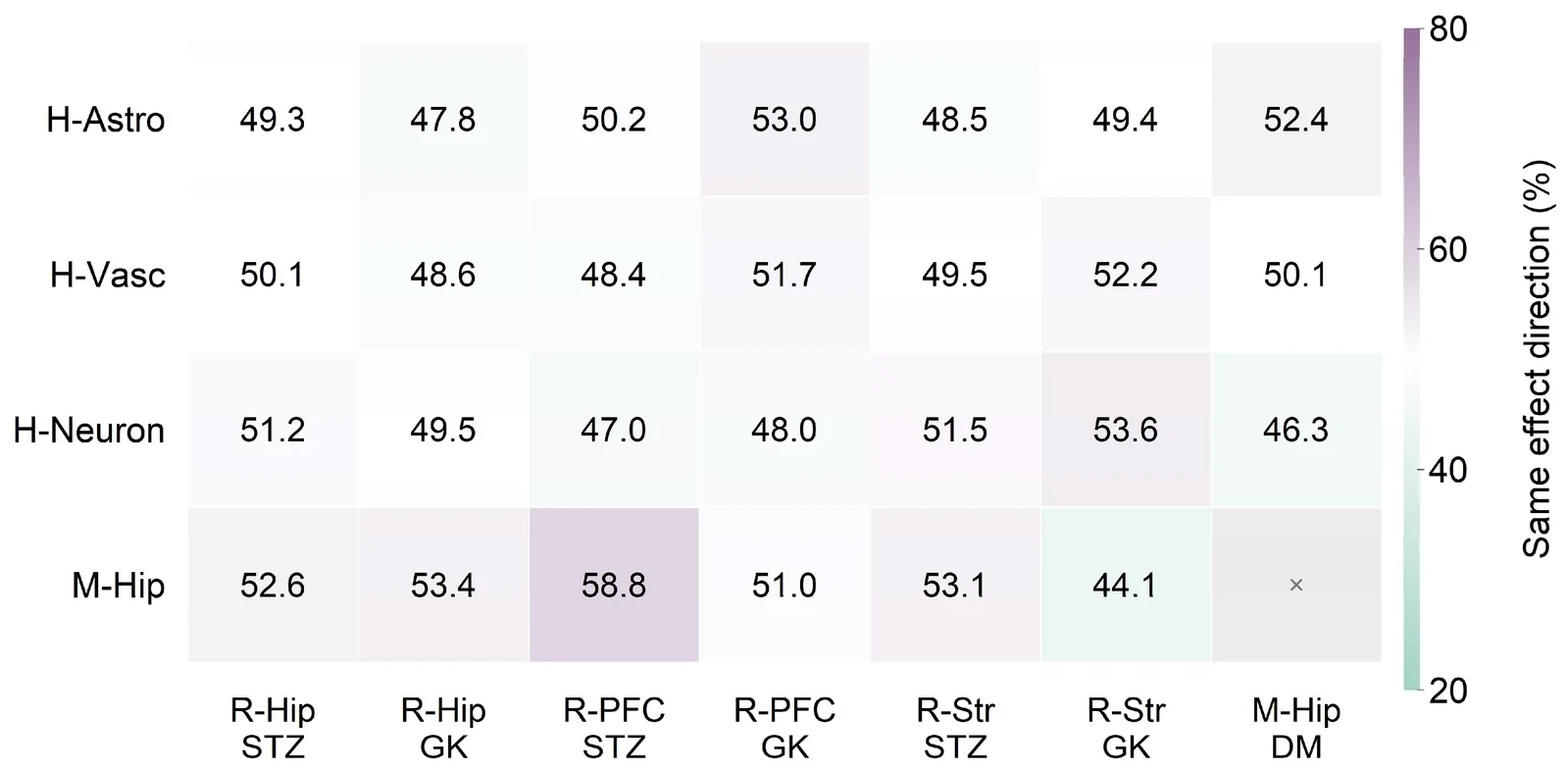
Cross-species agreement in disease-effect direction. Cells give percentages of common uniquely mapped genes with coefficients of the same sign. Purple denotes greater and green lower agreement, with white at 50% and color limits 20–80%. Printed values are percentages; a gray cross marks the unperformed mouse self-comparison. H-Astro, H-Vasc, H-Neuron: human astrocyte-, vascular-, and neuron-enriched fractions; R-Hip, R-PFC, R-Str: rat hippocampus, prefrontal cortex, and striatum; M-Hip, mouse hippocampus; STZ, streptozotocin; GK, Goto–Kakizaki; DM, diabetes. Agreement is descriptive.

**Figure 7:**
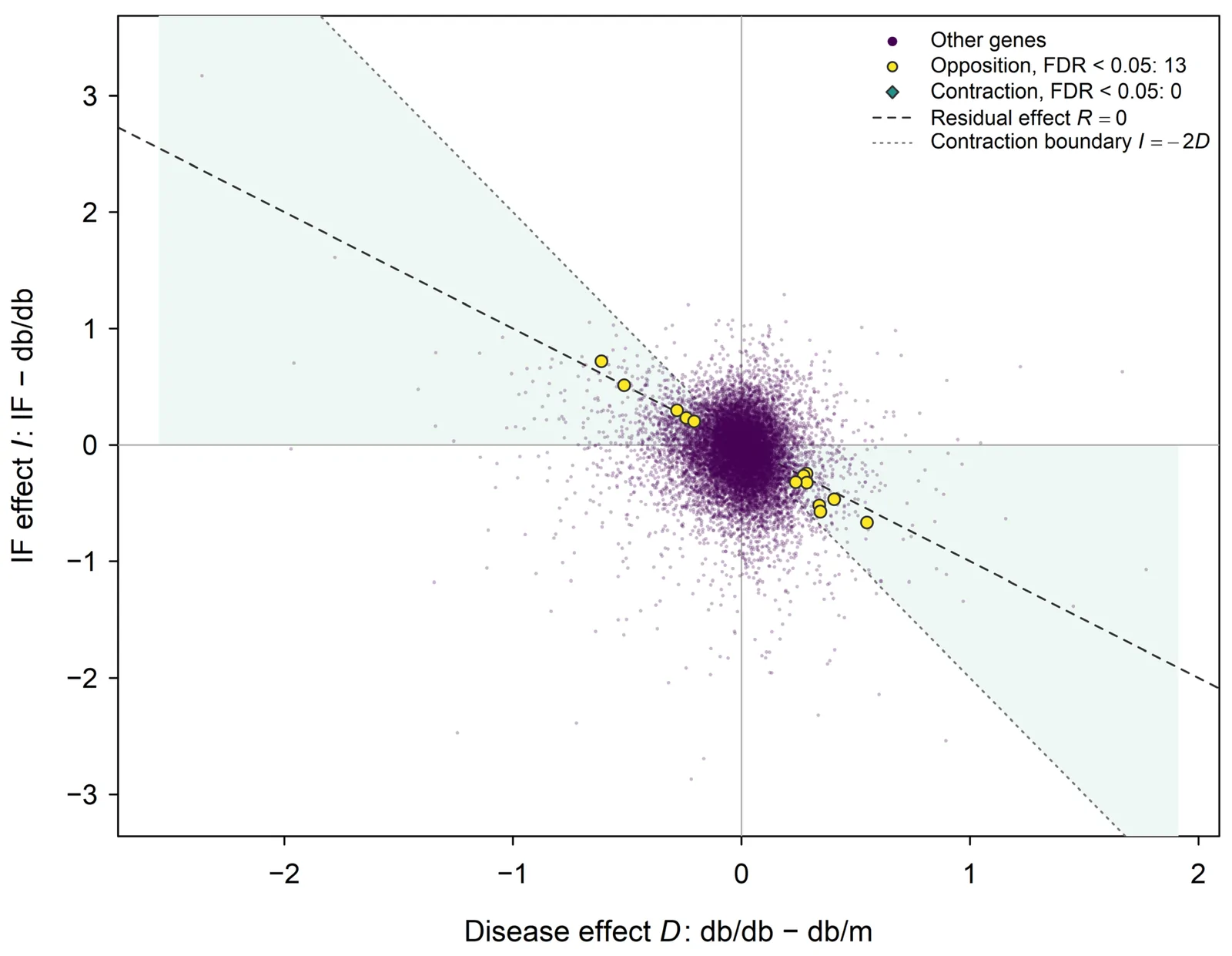
Disease and fasting effects in diabetic mouse hippocampus. *D* is db/db minus db/m, *I* is db/db mice subjected to IF minus untreated db/db mice, and *R* = *D* + *I* is db/db mice subjected to IF minus db/m. Effects are differences in mean log_2_(FPKM + 1). The pale green region satisfies |*R*| < |*D*| at the point estimates; the dashed line marks *R* = 0 and the dotted line marks *I* = −2*D*. Purple circles represent other tested genes, yellow circles the 13 genes meeting joint-family opposition FDR < 0.05, and green diamonds contraction discoveries, of which there were none. Gray horizontal and vertical lines mark zero IF and disease effects, respectively. Opposition requires disease and IF effects of opposite signs; contraction additionally requires a smaller absolute residual effect. The shaded region is descriptive, not a confidence region or an equivalence region. IF, intermittent fasting; FPKM, fragments per kilobase of transcript per million mapped fragments; FDR, false discovery rate.

**Figure 8:**
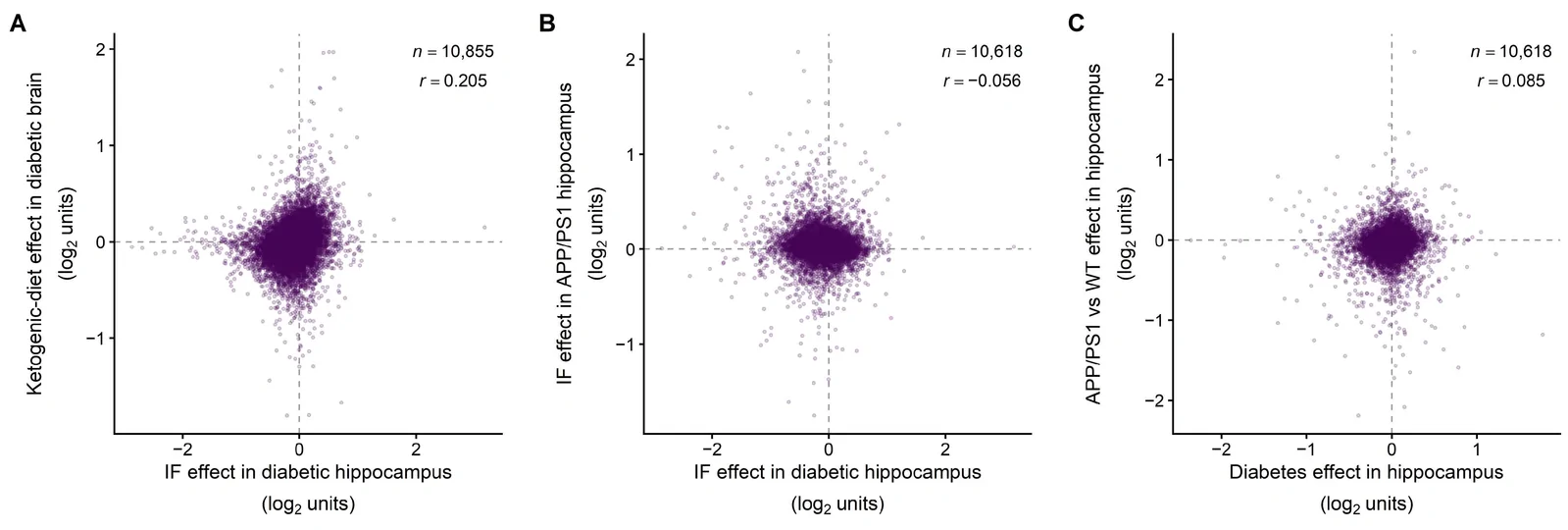
Concordance of effects across all shared genes in the mouse experiments. Panel (**A**) compares IF in db/db hippocampus with ketogenic diet in db/db whole brain (10,855 common genes). Panel (**B**) compares IF effects in diabetic and APP/PS1 hippocampus, and panel (**C**) compares their disease effects (10,618 common genes in each). Each purple point represents one common unambiguous gene symbol; dashed lines mark zero effects. Positive values indicate higher expression in the first named group. Displayed *r* values are coefficient Pearson correlations; Spearman correlations of moderated *t* statistics are reported separately in Table 3. Axes retain source scales: log_2_(FPKM + 1) differences for hippocampal FPKM data and voom effects on the log_2_ count scale for ketogenic diet. IF, intermittent fasting; WT, wild-type; APP/PS1, amyloid precursor protein/presenilin-1 model; FPKM, fragments per kilobase of transcript per million mapped fragments; *n*, common-gene count. The comparator for ketogenic diet is standard-fed db/db mice. Panel A compares different brain sampling schemes and dietary protocols; its correlation and shared genes cannot isolate an effect specific to diet from regional cell composition or other design differences.

**Figure 9:**
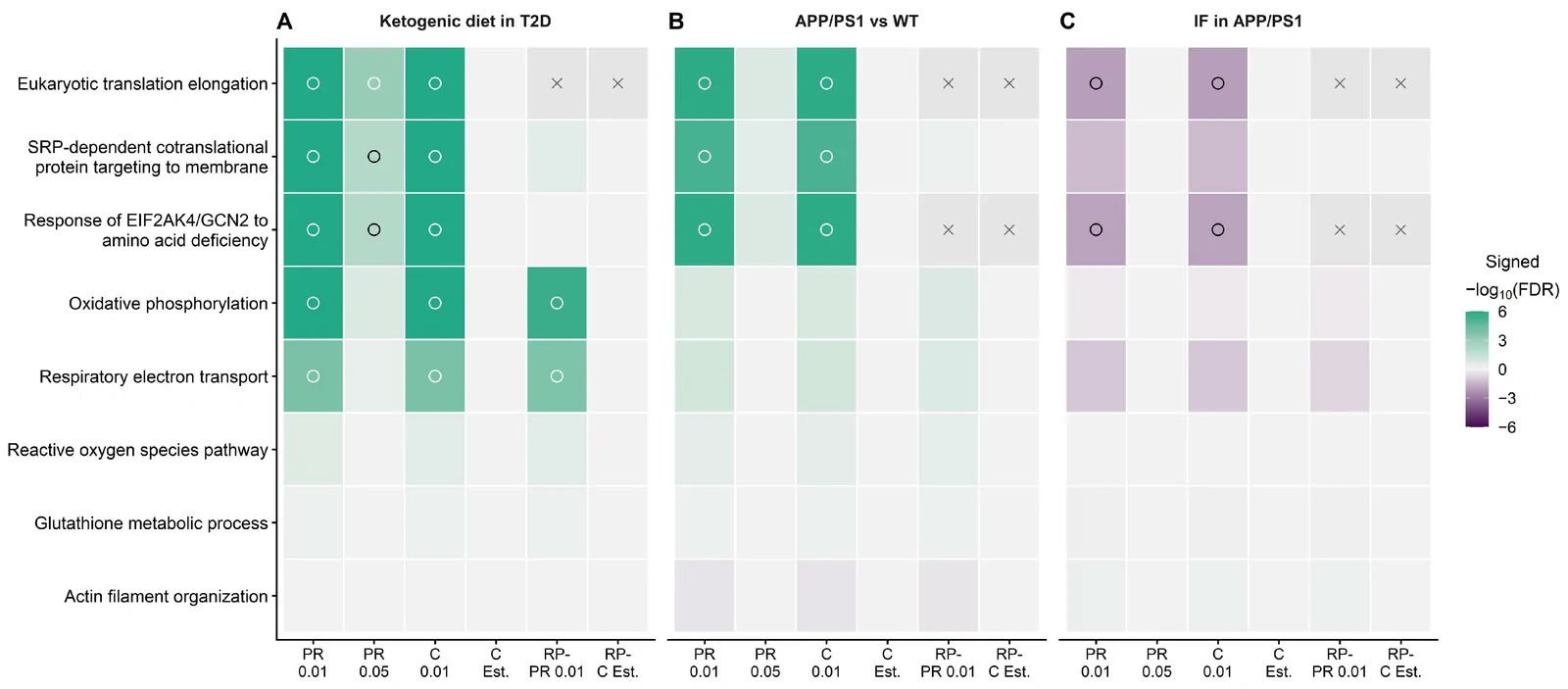
Selected-process evidence across dietary and disease contexts. Panels (**A**–**C**) show ketogenic diet versus standard diet in db/db whole brain, APP/PS1 versus wild-type under ad libitum feeding, and IF versus ad libitum in APP/PS1 hippocampus. Color shows competitive direction multiplied by −log_10_(per-contrast FDR), capped at magnitude six; positive scores are green and negative scores purple. Open circles mark FDR < 0.05; unmarked colored cells are tested nonsignificant results, and gray cells with crosses are unavailable or ineligible sets. FDR, false discovery rate; IF, intermittent fasting; APP/PS1, amyloid precursor protein/presenilin-1 model; PR, CAMERA-PR; C, CAMERA applied to expression matrices; Est., estimated correlation; RP-, exclusion of canonical cytosolic ribosomal protein large/small-subunit (RPL/RPS) genes; numeric labels, fixed intergene correlation; T2D, type 2 diabetes; WT, wild-type; SRP, signal recognition particle; EIF2AK4/GCN2, eukaryotic translation initiation factor 2 alpha kinase 4/general control nonderepressible 2. The eight processes are exploratory selections, and scores do not measure biochemical activity.

**Figure 10:**
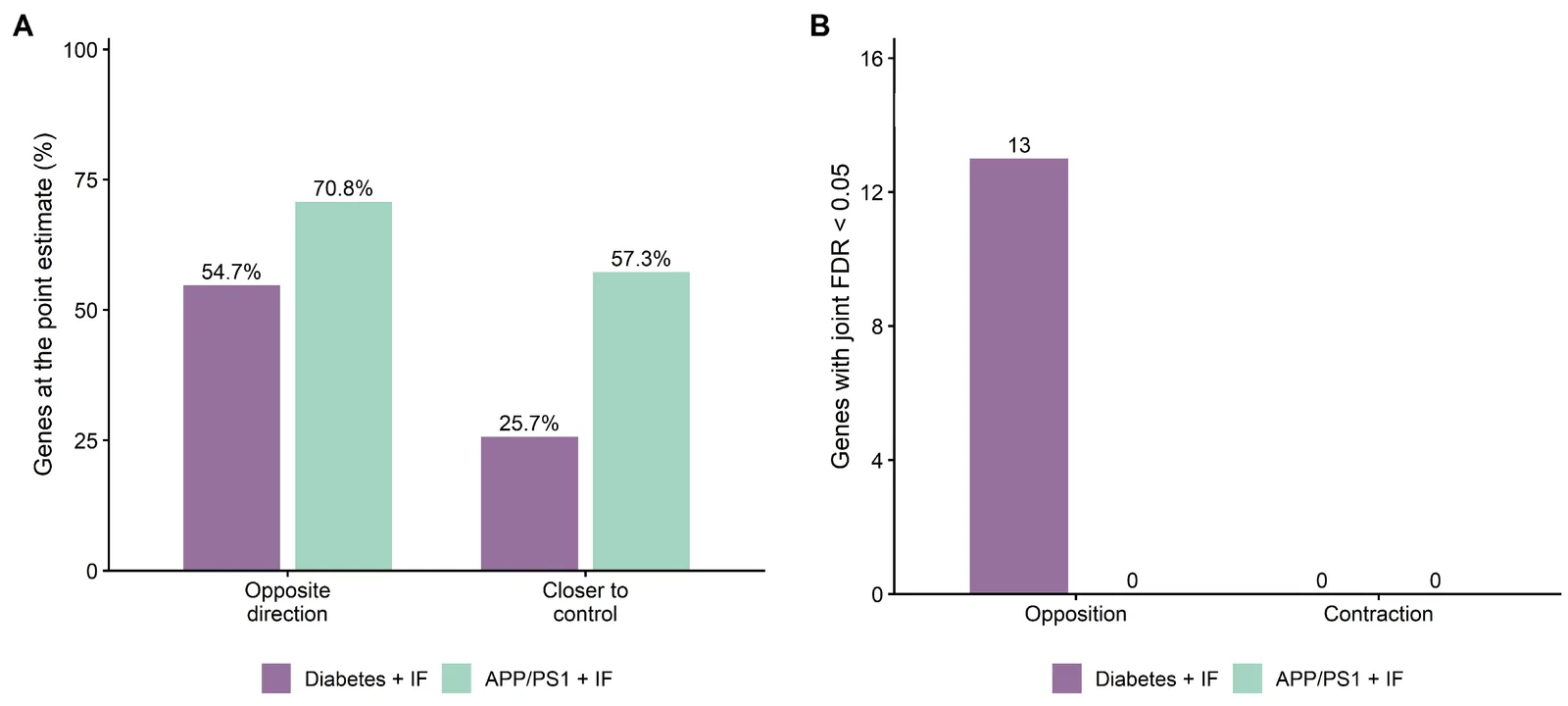
Descriptive response geometry and inference incorporating contrast covariance in diabetic and APP/PS1 hippocampus. Panel (**A**) shows percentages of all tested genes with opposite disease and IF point estimates or a smaller residual distance from the designated control. Opposite direction means *D* and *I* have opposite signs; closer to control means |*D* + *I*| < |*D*|, where *D* is disease minus control and *I* is IF minus untreated disease. Panel (**B**) shows significant gene counts under joint-family conjunction FDR < 0.05. Universes comprise 13,237 diabetic and 12,294 corrected APP/PS1 genes. Purple denotes the diabetes-plus-IF experiment and green the APP/PS1-plus-IF experiment. The controls are db/m and wild-type ad libitum, respectively. Shared untreated disease groups induce contrast covariance, which is retained in inference. The descriptive percentages in panel (**A**) do not establish restoration or equivalence to control expression; no primary contraction test was significant. APP/PS1, amyloid precursor protein/presenilin-1 model; IF, intermittent fasting; FDR, false discovery rate.

**Table 1:** Supported opposition genes in diabetic mouse hippocampus.

Gene	*D* (95% CI)	*I* (95% CI)	*R* (95% CI)	Opposition FDR	Contraction FDR
*Abhd16a*	−0.282 (−0.372, −0.192)	0.297 (0.205, 0.389)	0.015 (−0.075, 0.105)	0.01221	0.208451
*Neurod6*	0.549 (0.360, 0.738)	−0.666 (−0.859, −0.472)	−0.116 (−0.306, 0.073)	0.022695	0.575232
*Hnrnpa0*	0.272 (0.178, 0.366)	−0.265 (−0.360, −0.169)	0.007 (−0.086, 0.101)	0.02396	0.201942
*Cacnb2*	0.340 (0.216, 0.465)	−0.521 (−0.648, −0.394)	−0.180 (−0.305, −0.056)	0.02396	1
*Cryba4*	−0.613 (−0.841, −0.385)	0.718 (0.485, 0.951)	0.105 (−0.123, 0.333)	0.02396	0.607106
*Lrpap1*	−0.241 (−0.327, −0.156)	0.232 (0.145, 0.319)	−0.009 (−0.095, 0.076)	0.02396	0.214795
*Plk2*	0.285 (0.194, 0.375)	−0.246 (−0.338, −0.153)	0.039 (−0.051, 0.130)	0.02396	0.092902
*Gpr22*	0.406 (0.252, 0.559)	−0.468 (−0.625, −0.310)	−0.062 (−0.216, 0.092)	0.02396	0.60056
*Zfpm1*	0.286 (0.177, 0.395)	−0.324 (−0.436, −0.213)	−0.038 (−0.147, 0.071)	0.02396	0.579799
*Cpne8*	0.345 (0.213, 0.478)	−0.573 (−0.708, −0.438)	−0.228 (−0.360, −0.096)	0.02396	1
*Tomm40L*	−0.207 (−0.286, −0.128)	0.202 (0.122, 0.282)	−0.005 (−0.084, 0.074)	0.03919	0.333977
*Slc24a3*	0.238 (0.141, 0.334)	−0.320 (−0.419, −0.221)	−0.082 (−0.179, 0.015)	0.049544	1
*Myo1a*	−0.513 (−0.719, −0.308)	0.514 (0.303, 0.724)	0.000 (−0.205, 0.206)	0.049561	0.454821

*D*, db/db minus db/m; *I*, db/db mice subjected to IF minus untreated db/db mice; *R* = *D* + *I*. Effects are differences in mean log_2_(FPKM + 1); CI, pointwise moderated confidence interval; FDR, BH adjustment jointly across 26,474 opposition and contraction tests. Intervals are pointwise, not simultaneous.

**Table 2:** Primary results in the additional dietary datasets.

Contrast	*n*	Genes	Gene Hits (Higher/Lower)	Sets	PR *ρ* 0.01	PR *ρ* 0.05	C *ρ* 0.01	C Est.
KD/standard db/db	8	14,895	999 (521/478)	4923	116	16	116	0
AD/WT, AL	55	12,294	74 (36/38)	4148	28	0	28	0
IF/AL, AD	55	12,294	0 (0/0)	4148	3	0	3	0
AD-IF/WT-AL	55	12,294	17 (11/6)	4148	0	0	0	0
IF/AL, WT	55	12,294	0 (0/0)	4148	0	0	0	0
Genotype × IF	55	12,294	0 (0/0)	4148	0	0	0	0
AD/WT, IF	55	12,294	4 (4/0)	4148	16	0	16	0

Gene hits use per-contrast BH FDR < 0.05. Pathway hits use per-contrast BH FDR < 0.05 over the eligible collections. KD, ketogenic diet; AD, APP/PS1 genotype; WT, wild-type; AL, ad libitum; IF, intermittent fasting; PR, CAMERA-PR; C, CAMERA applied to expression matrices; Est., estimated correlation; ρ, intergene correlation. The KD comparator is standard-fed db/db, not a healthy group. AD contrasts use the corrected 12,294-feature universe. The auxiliary 2*D* + *I* contrast is omitted from biological discovery summaries.

**Table 3:** Concordance across the mouse experiments.

Pair	Common Genes	Coefficient Pearson *r*	Moderated-*t* Spearman *ρ*	Same Sign (%)	Both Gene FDR < 0.05	Common Sets	Both Set FDR < 0.05	Same-Direction Significant Sets
Diabetic IF/ KD	10,855	0.205064	0.283749	59.954	447	4214	59	59
Diabetic IF/ AD IF	10,618	−0.055716	0.015047	45.837	0	4074	0	0
Diabetes/ AD	10,618	0.085432	0.207823	56.442	9	4074	4	4

All common unambiguous symbols were retained without selecting genes by statistical significance. Gene overlaps use BH FDR < 0.05 within each source contrast. Of the 447 genes significant in both diabetic dietary experiments, 359 had matching effect directions and 88 had opposite directions; six of the nine genes significant in both disease comparisons had matching directions. Pathway overlap uses CAMERA-PR at a fixed correlation of 0.01 and includes related sets, not independent mechanisms. No shared pathway had external estimated-correlation support. IF, intermittent fasting; KD, ketogenic diet; AD, APP/PS1 model; FDR, false discovery rate.

**Figure A1:**
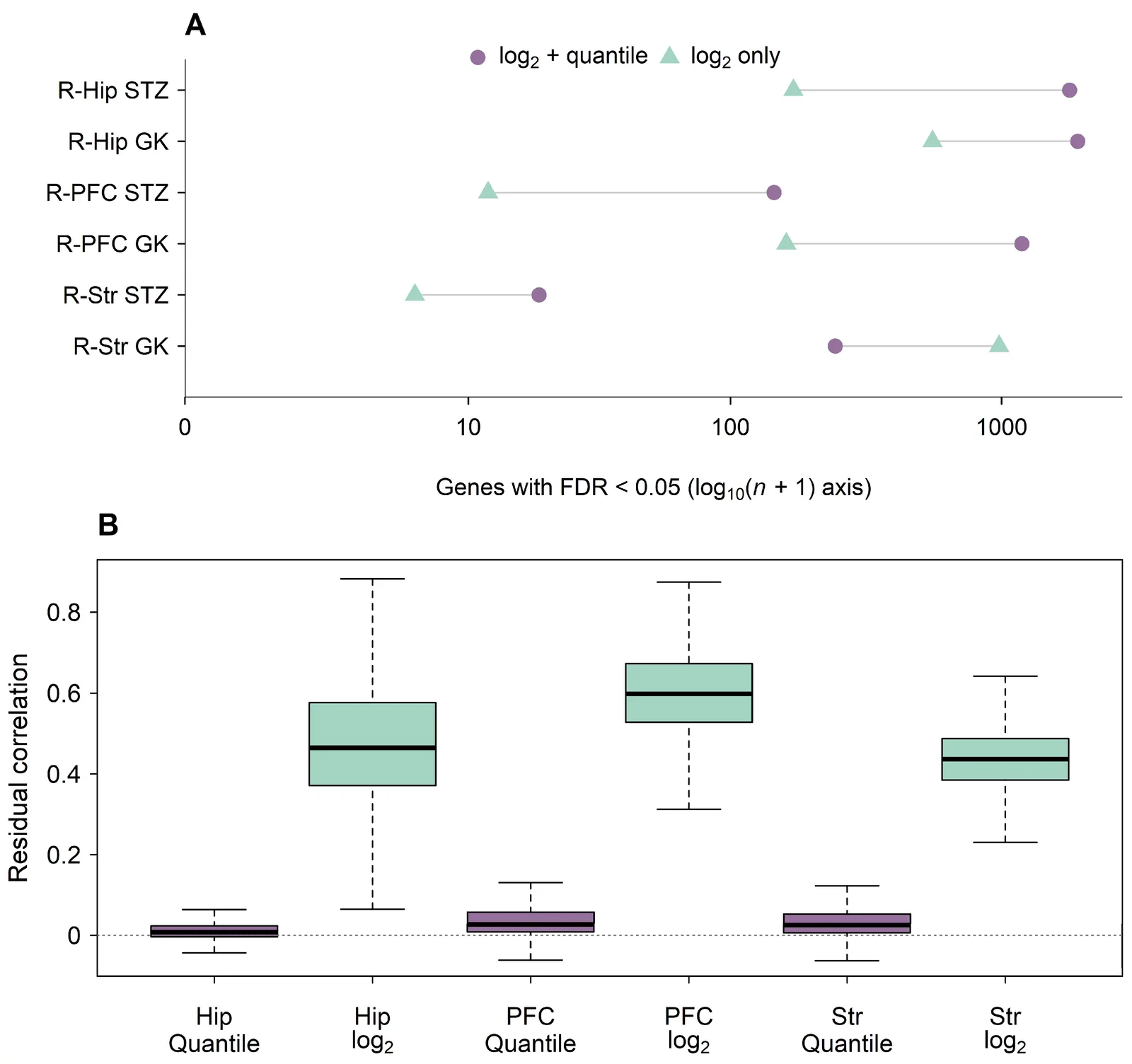
Rat normalization sensitivity. Panel (**A**) compares gene discovery counts after log_2_ transformation with regional quantile normalization (purple circles) and log_2_ transformation only (green triangles). Gray segments connect the two preprocessing results for each contrast. The horizontal axis is log_10_(*n* + 1), with ticks labeled by gene counts. Panel (**B**) shows residual correlations within tested gene sets after fitting the group design. Purple and green boxes denote quantile-normalized and log_2_-only inputs. Center lines are medians; boxes span the interquartile range (IQR), and whiskers extend to the most extreme observations within 1.5 IQR of the quartiles. Outliers are omitted; the dotted line marks zero correlation. Correlations describe overlapping gene sets, not independent observations. R, rat; STZ, streptozotocin; GK, Goto–Kakizaki; Hip, hippocampus; PFC, prefrontal cortex; Str, striatum; FDR, false discovery rate. Each regional group contained three pools.

**Figure A2:**
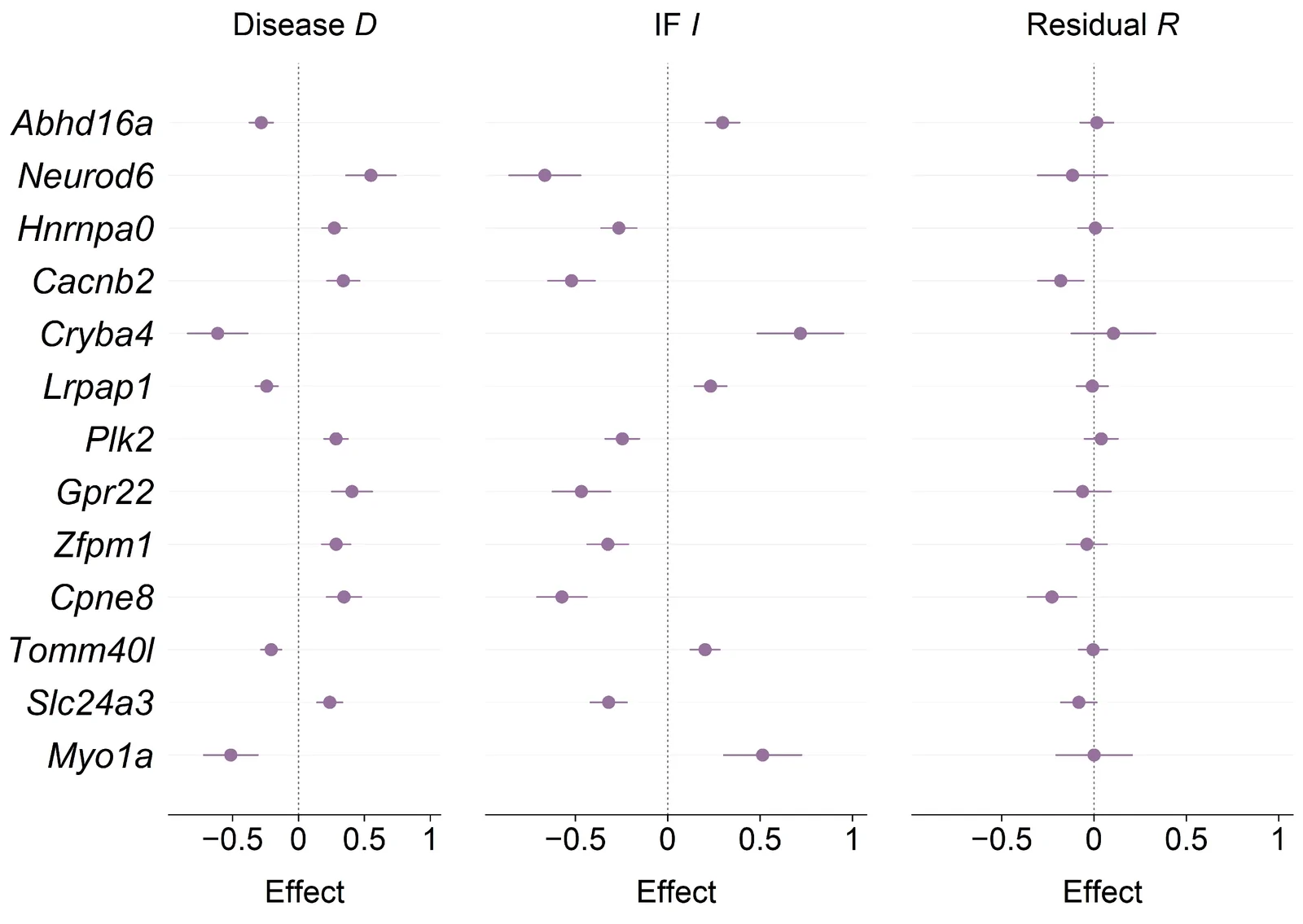
Effects and pointwise 95% moderated confidence intervals for the 13 genes meeting joint-family opposition FDR < 0.05 in diabetic mouse hippocampus. *D*, disease effect (db/db minus db/m); *I*, IF effect (db/db mice subjected to IF minus untreated db/db mice); *R*, residual effect (db/db mice subjected to IF minus db/m). Purple points and intervals show differences in mean log_2_(FPKM + 1); pale horizontal lines assist matching genes across panels and dotted vertical lines mark zero effects. FDR, false discovery rate; IF, intermittent fasting; FPKM, fragments per kilobase of transcript per million mapped fragments. Intervals are pointwise, not simultaneous.

**Figure A3:**
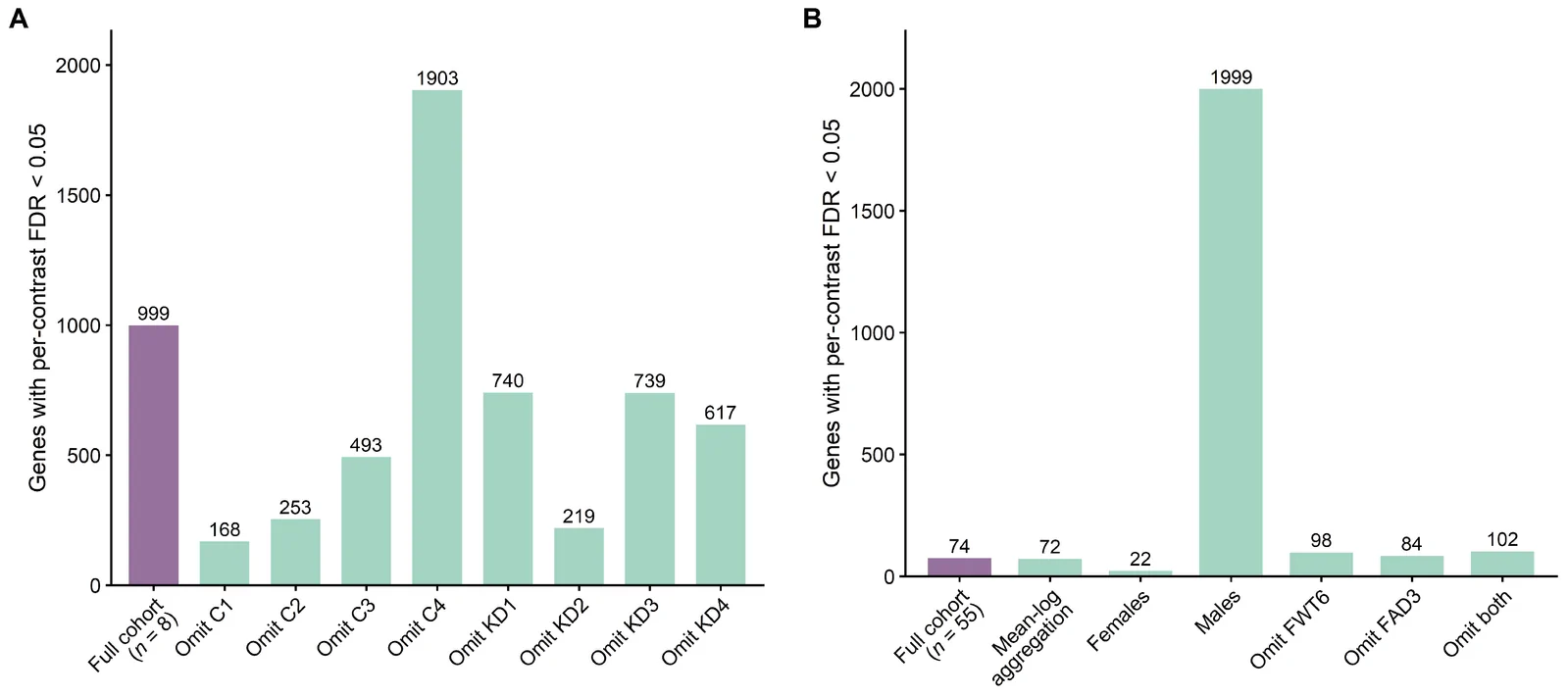
Gene-discovery sensitivity in the additional dietary datasets. Panel (**A**) shows ketogenic diet versus standard diet in GSE249467 for the full cohort of eight mice and each leave-one-out fit. Panel (**B**) shows APP/PS1 versus wild-type under ad libitum feeding in corrected GSE164461 for the full cohort of 55 mice, mean aggregation, sex strata, and QC omissions. Purple denotes the primary full-cohort model and green sensitivity models. Counts use within-model and within-contrast BH FDR < 0.05. Numbers above bars are gene counts; *n* is the total number of mice included in the full-cohort fit, across all groups. Omit indicates refitting after removing the named sample; C1–C4 are standard-diet controls and KD1–KD4 ketogenic-diet mice. Mean-log aggregation averages transformed expression across rows assigned to the same gene; the primary model selects the highest-mean row. BH, Benjamini–Hochberg; FDR, false discovery rate; APP/PS1, amyloid precursor protein/presenilin-1 model; KD, ketogenic diet; QC, quality control; FWT6, flagged female wild-type sample; FAD3, flagged female APP/PS1 sample. Panels have separate *y*-axis scales; sex-stratum counts are not an interaction test.

**Figure A4:**
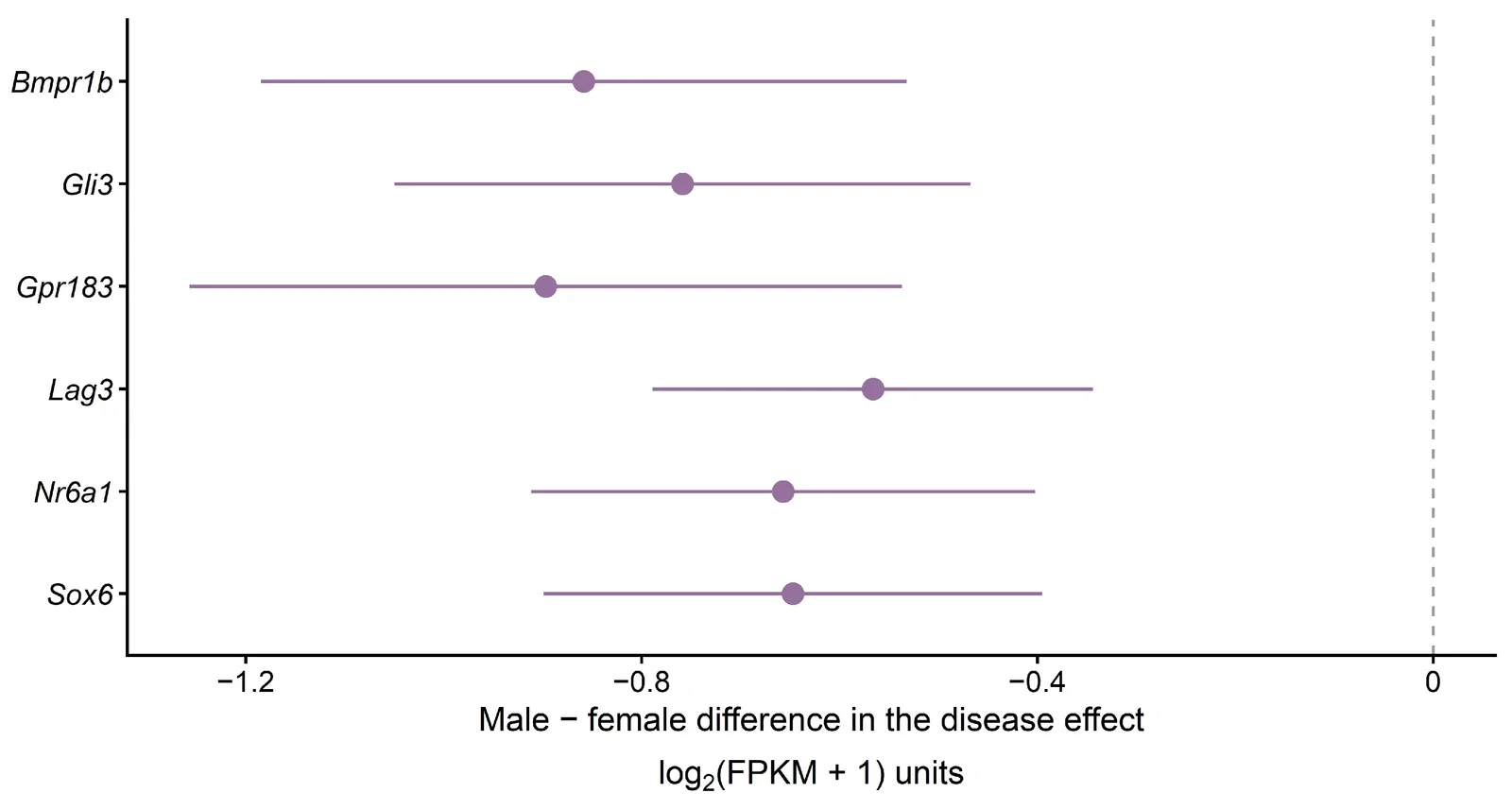
Exploratory disease-by-sex modifiers in corrected APP/PS1 hippocampus. Purple circles show male-minus-female disease coefficients and horizontal lines their pointwise 95% moderated confidence intervals for six genes meeting joint FDR < 0.05 across 36,882 sex-modifier tests. The disease effect is APP/PS1 minus wild-type under ad libitum feeding. Negative coefficients indicate a more negative, or less positive, disease effect in males. Effects are measured on the log_2_(FPKM + 1) scale. The dashed vertical line marks no male–female difference. APP/PS1, amyloid precursor protein/presenilin-1 model; FPKM, fragments per kilobase of transcript per million mapped fragments; FDR, false discovery rate. Intervals are pointwise, not simultaneous.

## Data Availability

The transcriptomic datasets analyzed in this study are publicly available through the NCBI Gene Expression Omnibus (GEO) under accession numbers GSE161355, GSE34451, GSE125387, GSE249467, and GSE164461. Complete analysis output tables, input manifests, annotation records, and the R scripts developed for this study are available from the corresponding author upon reasonable request.

## Abbreviations

The following abbreviations are used in this manuscript:

AD	Alzheimer’s disease
AL	Ad libitum
APP/PS1	Amyloid precursor protein/presenilin-1
ATP	Adenosine triphosphate
BH	Benjamini–Hochberg procedure
CAMERA	Correlation-Adjusted MEan RAnk gene set test
CAMERA-PR	CAMERA for pre-ranked gene statistics
CEL	Affymetrix cell intensity file
CI	Confidence interval
DM	Diabetes mellitus
EIF2AK4/GCN2	Eukaryotic translation initiation factor 2 alpha kinase 4/general control nonderepressible 2
FDR	False discovery rate
FPKM	Fragments per kilobase of transcript per million mapped fragments
GEO	Gene Expression Omnibus
GK	Goto–Kakizaki
GO	Gene Ontology
IF	Intermittent fasting
IGF-1	Insulin-like growth factor 1
IQR	Interquartile range
KD	Ketogenic diet
MRPL/MRPS	Mitochondrial ribosomal proteins, large/small subunit
MSigDB	Molecular Signatures Database
NCBI	National Center for Biotechnology Information
NLRP3	NLR family pyrin domain containing 3
QC	Quality control
RNA-seq	RNA sequencing
RPL/RPS	Ribosomal proteins, large/small subunit
RPS6 kinases	Ribosomal protein S6 kinases
SARS-CoV-2	Severe acute respiratory syndrome coronavirus 2
SRP	Signal recognition particle
STZ	Streptozotocin
T2D	Type 2 diabetes
WT	Wild-type

## Appendix A

**Table A1:** Rat gene discoveries under two normalization choices.

Comparison	Genes Tested	log_2_ + Quantile	log_2_ Only
R-Hip: STZ/ctrl	17,268	1778	171
R-Hip: GK/ctrl	17,268	1905	557
R-PFC: STZ/ctrl	17,268	145	12
R-PFC: GK/ctrl	17,268	1187	161
R-Str: STZ/ctrl	17,268	19	6
R-Str: GK/ctrl	17,268	244	979

Counts use per-contrast BH FDR < 0.05. Each group contained three pools; quantile normalization was applied within regions. Gene representation and contrasts were otherwise matched. The alternatives assess dependence on preprocessing, not superiority of a method. R-Hip, rat hippocampus; R-PFC, rat prefrontal cortex; R-Str, rat striatum; STZ, streptozotocin; GK, Goto–Kakizaki; ctrl, control.

**Table A2:** APP/PS1 IF genes under two BH testing families.

Gene	Raw *p*	IF Rank	Combined Rank	Per-Contrast BH FDR	Combined BH FDR
*Kdm6b*	0.0001121905	1	227	0.607006	0.043818
*Prkar2a*	0.0001151887	2	231	0.607006	0.043838
*Myo16*	0.0001481226	3	269	0.607006	0.048819

The contrast is IF minus ad libitum within APP/PS1 mice in the corrected reference model. The per-contrast family contains 12,294 tests; the combined family contains 88,659 tests across seven biological contrasts from GSE164461 and GSE249467. Ranks refer to ascending raw *p* values in the complete respective family. BH adjustment uses the entire ordered distribution, so an adjusted value need not equal the number of tests multiplied by the raw *p* value divided by its own rank. Raw *p* values are rounded to ten decimal places and FDR values to six. The combined family does not provide separate FDR control for the IF subset.

**Table A3:** Gene discovery sensitivity in the additional dietary experiments.

Model	*n*	Disease	Diet in Disease	Res.	WT IF	Genotype × IF	Disease Under IF
KD full cohort	8	NA	999	NA	NA	NA	NA
KD omit C1	7	NA	168	NA	NA	NA	NA
KD omit C2	7	NA	253	NA	NA	NA	NA
KD omit C3	7	NA	493	NA	NA	NA	NA
KD omit C4	7	NA	1903	NA	NA	NA	NA
KD omit KD1	7	NA	740	NA	NA	NA	NA
KD omit KD2	7	NA	219	NA	NA	NA	NA
KD omit KD3	7	NA	739	NA	NA	NA	NA
KD omit KD4	7	NA	617	NA	NA	NA	NA
AD full cohort	55	74	0	17	0	0	4
AD mean aggregation	55	72	0	16	0	0	4
AD females	27	22	1	35	0	0	31
AD males	28	1999	23	185	0	0	0
AD omit FWT6	54	98	0	20	0	0	4
AD omit FAD3	54	84	0	19	0	0	4
AD omit both	53	102	0	21	0	0	4

Counts use BH FDR < 0.05 within each model and contrast. All KD models retained 14,895 genes; all corrected AD models retained 12,294. Disease: APP/PS1 AL minus WT AL; diet in disease: KD minus standard diet within db/db, or AD IF minus AD AL; Res., residual: AD IF minus WT AL; WT IF: WT IF minus WT AL; disease under IF: AD IF minus WT IF. The auxiliary 2*D* + *I* contrast is not a biological discovery endpoint. *n* is total mice in the fit. Sex-stratum sample counts are 27 females and 28 males. KD, ketogenic diet; AD, APP/PS1; WT, wild-type; AL, ad libitum; IF, intermittent fasting; NA, contrast unavailable.

Rat filtering and normalization against control samples from the source study were not reconstructed from raw arrays. In the AD annotation audit, 14,100 rows lacked gene symbols and 1520 contained assignments to distinct symbols that remained unresolved. Appendix A Table A4 documents these assignments, the five recovered expressed genes, and the four tested annotation universes.

**Table A4:** AD annotation sensitivity and source coverage.

Representation	Features	Disease Gene Hits	IF Gene Hits	Residual Gene Hits	IF Pathway Hits	Minimum IF Gene FDR
Original	12,309	74	0	17	4	0.607775
Five symbols added	12,314	74	0	17	4	0.607955
Date labels removed	12,289	74	0	17	3	0.606826
Combined correction	12,294	74	0	17	3	0.607006

All representations used the same 55 samples and group-plus-sex design (50 residual degrees of freedom). Gene hits use per-contrast BH FDR < 0.05; pathway hits use CAMERA-PR at a fixed correlation of 0.01. Coefficients were unchanged on shared features; no common-gene discoveries were gained or lost, and Spearman correlations of moderated *t* statistics exceeded 0.99999999 in all comparisons. Joint opposition and contraction discoveries were zero in every representation. Recovery resolved identical repetitions in 193 source rows and added *Gm22*, *Gm29695*, *Gm34354*, *Pate2*, and *Zfp618* after expression filtering. Twenty original tested date labels were removed without reassignment. Of 56,090 source rows, 14,100 missing-symbol rows and 1520 distinct-symbol assignments remained unresolved. AD, APP/PS1; IF, intermittent fasting; FDR, false discovery rate.

**Table A5:** Primary pathway results across methods and gene universes.

Contrast	Method	Input	Eligible Sets	Hits	Minimum FDR
AD/WT, AL	PR 0.01	All	4148	28	1.63 × 10^−6^
AD/WT, AL	PR 0.05	All	4148	0	0.165896
AD/WT, AL	C 0.01	All	4148	28	1.65 × 10^−6^
AD/WT, AL	C estimated	All	4148	0	0.999688
IF/AL, AD	PR 0.01	All	4148	3	0.008874
IF/AL, AD	PR 0.05	All	4148	0	0.997829
IF/AL, AD	C 0.01	All	4148	3	0.008923
IF/AL, AD	C estimated	All	4148	0	0.999866
AD-IF/WT-AL	PR 0.01	All	4148	0	0.0553
AD-IF/WT-AL	PR 0.05	All	4148	0	0.99896
AD-IF/WT-AL	C 0.01	All	4148	0	0.0558
AD-IF/WT-AL	C estimated	All	4148	0	0.99998
IF/AL, WT	PR 0.01	All	4148	0	0.944082
IF/AL, WT	PR 0.05	All	4148	0	0.999884
IF/AL, WT	C 0.01	All	4148	0	0.944635
IF/AL, WT	C estimated	All	4148	0	0.999984
Genotype × IF	PR 0.01	All	4148	0	0.465902
Genotype × IF	PR 0.05	All	4148	0	0.99969
Genotype × IF	C 0.01	All	4148	0	0.464909
Genotype × IF	C estimated	All	4148	0	1
AD/WT, IF	PR 0.01	All	4148	16	8.38 × 10^−4^
AD/WT, IF	PR 0.05	All	4148	0	0.999885
AD/WT, IF	C 0.01	All	4148	16	8.49 × 10^−4^
AD/WT, IF	C estimated	All	4148	0	1
AD/WT, AL	PR 0.01	RP-	4138	0	0.144585
AD/WT, AL	PR 0.05	RP-	4138	0	0.995598
AD/WT, AL	C 0.01	RP-	4138	0	0.145099
AD/WT, AL	C estimated	RP-	4138	0	0.999956
IF/AL, AD	PR 0.01	RP-	4138	0	0.228971
IF/AL, AD	PR 0.05	RP-	4138	0	0.99871
IF/AL, AD	C 0.01	RP-	4138	0	0.227996
IF/AL, AD	C estimated	RP-	4138	0	0.999753
AD-IF/WT-AL	PR 0.01	RP-	4138	0	0.111075
AD-IF/WT-AL	PR 0.05	RP-	4138	0	0.999052
AD-IF/WT-AL	C 0.01	RP-	4138	0	0.112117
AD-IF/WT-AL	C estimated	RP-	4138	0	0.999911
IF/AL, WT	PR 0.01	RP-	4138	0	0.94685
IF/AL, WT	PR 0.05	RP-	4138	0	0.999964
IF/AL, WT	C 0.01	RP-	4138	0	0.947737
IF/AL, WT	C estimated	RP-	4138	0	0.999914
Genotype × IF	PR 0.01	RP-	4138	0	0.486515
Genotype × IF	PR 0.05	RP-	4138	0	0.999146
Genotype × IF	C 0.01	RP-	4138	0	0.485341
Genotype × IF	C estimated	RP-	4138	0	0.999844
AD/WT, IF	PR 0.01	RP-	4138	0	0.183452
AD/WT, IF	PR 0.05	RP-	4138	0	1
AD/WT, IF	C 0.01	RP-	4138	0	0.184509
AD/WT, IF	C estimated	RP-	4138	0	0.999966
KD/standard db/db	PR 0.01	All	4923	116	1.42 × 10^−14^
KD/standard db/db	PR 0.05	All	4923	16	9.82 × 10^−4^
KD/standard db/db	C 0.01	All	4923	116	1.59 × 10^−14^
KD/standard db/db	C estimated	All	4923	0	0.999021
KD/standard db/db	PR 0.01	RP-	4916	87	2.59 × 10^−6^
KD/standard db/db	PR 0.05	RP-	4916	0	0.168767
KD/standard db/db	C 0.01	RP-	4916	89	2.58 × 10^−6^
KD/standard db/db	C estimated	RP-	4916	0	0.99645

PR, CAMERA-PR; C, rank CAMERA applied to expression matrices; numeric values, fixed intergene correlation; estimated, residual correlation estimated separately for each gene set; All, complete measured universe; RP-, exclusion of canonical cytosolic RPL/RPS genes. Hits use per-contrast BH FDR < 0.05 across the eligible collections. AD, APP/PS1; WT, wild-type; AL, ad libitum; IF, intermittent fasting; KD, ketogenic diet. Unavailable sets are excluded from eligibility, not counted as nonsignificant tests. Complete per-set statistics are retained in machine-readable project outputs.

**Table A6:** Selected dietary pathway FDR values.

Comparison and Process	PR *ρ* 0.01	C *ρ* 0.01	PR *ρ* 0.05	RP- PR *ρ* 0.01	C Estimated
KD: oxidative phosphorylation	2.65 × 10^−7^	2.64 × 10^−7^	0.162203	2.59 × 10^−6^	0.999021
KD: respiratory electron transport	1.06 × 10^−4^	1.09 × 10^−4^	0.515078	1.58 × 10^−4^	0.999021
AD IF: translation elongation	0.008874	0.008923	0.997829	NA	0.999866
AD IF: EIF2AK4/GCN2 response	0.01193	0.011977	0.997829	NA	0.999866
AD IF: host translation machinery	0.047991	0.047878	0.997829	NA	0.999866
AD IF: SRP targeting	0.050885	0.051048	0.997829	0.959347	0.999866

Every entry is the within-contrast BH FDR over its complete eligible collection. KD, ketogenic diet versus standard diet in db/db; AD IF, IF versus ad libitum in APP/PS1; PR, CAMERA-PR; C, CAMERA applied to expression matrices; RP-, canonical cytosolic RPL/RPS exclusion; ρ, intergene correlation; SRP, signal recognition particle. The two KD respiratory sets had Up direction; the four AD IF sets had Down direction at baseline PR ρ 0.01. The host-translation set corresponds to the Reactome pathway “SARS-CoV-2 modulates host translation machinery”; this name does not imply infection in the analyzed samples. NA denotes a set that became ineligible after exclusion.

**Table A7:** Corrected AD response geometry across model specifications.

Model	Opposite Points	Opposite (%)	Closer Points	Closer (%)	Opp. Hits	Contr. Hits	Noise Correlation
AD full cohort	8700	70.766	7039	57.256	0	0	−0.498060
AD mean aggregation	8712	70.864	7046	57.313	0	0	−0.498060
AD females	7834	63.722	5872	47.763	0	0	−0.496139
AD males	8974	72.995	7772	63.218	0	0	−0.500000
AD omit FWT6	8725	70.970	7254	59.004	0	0	−0.490472
AD omit FAD3	8670	70.522	7045	57.304	0	0	−0.516859
AD omit both	8653	70.384	7219	58.720	0	0	−0.508983

All models retained 12,294 genes. *D* is AD AL minus WT AL; *I* is AD IF minus AD AL; *R* = *D* + *I*. Opposite and closer are descriptive point-estimate categories. Opp., opposition; Contr., contraction. Hits use BH FDR < 0.05 jointly across 24,588 opposition and contraction tests within each model. Noise correlation is the correlation between contrast sampling errors implied by the fitted model. It is distinct from the observed correlation across gene coefficients. AD, APP/PS1; WT, wild-type; AL, ad libitum; IF, intermittent fasting.

**Table A8:** Formal sex-modifier discovery counts.

Male-Minus-Female Modifier	Tests	Within-Modifier Hits	Joint-Family Hits	Minimum Joint FDR
IF effect in AD	12,294	0	0	0.06153
Disease effect	12,294	434	6	0.03868
IF effect in WT	12,294	0	0	0.076607

The joint family contained 36,882 tests across three modifiers. All contrasts used the corrected 12,294-feature universe and eight genotype-by-diet-by-sex group means, with 47 residual degrees of freedom. Hits use BH FDR < 0.05. Discovery counts within each sex are not tests of effect modification. AD, APP/PS1; WT, wild-type; IF, intermittent fasting.

**Table A9:** Supported disease-by-sex modifiers in APP/PS1 hippocampus.

Gene	Male–Female Coefficient	95% CI	*p* Value	Within-Modifier FDR	Joint-Family FDR
*Bmpr1b*	−0.858545	−1.184917, −0.532173	2.86 × 10^−6^	0.012893	0.03868
*Gli3*	−0.758685	−1.049949, −0.467420	3.43 × 10^−6^	0.012893	0.03868
*Gpr183*	−0.897051	−1.256931, −0.537172	7.47 × 10^−6^	0.015296	0.045889
*Lag3*	−0.566293	−0.788920, −0.343667	5.24 × 10^−6^	0.012893	0.03868
*Nr6a1*	−0.657020	−0.911519, −0.402522	4.03 × 10^−6^	0.012893	0.03868
*Sox6*	−0.647256	−0.899274, −0.395237	4.42 × 10^−6^	0.012893	0.03868

The modifier compares male and female APP/PS1-minus-WT effects under ad libitum feeding. Coefficients are differences in mean log_2_(FPKM + 1). CI, pointwise moderated confidence interval; FDR, false discovery rate; joint family, 36,882 tests across the three modifiers. Intervals are not simultaneous; no fasting efficacy inference follows from these disease modifiers.

**Table A10:** Human donor characteristics.

Case	Group	Sex	Age (Years)	PMI (Hours)	Brain pH	Braak Stage	Dementia at Death
C1	Control	female	87	60	6.1	II	no
C2	Control	male	92	33	6.8	II	yes
C3	Control	male	74	NR	6.3	II	no
C4	Control	male	75	29	7.3	0	no
C5	Control	female	73	20	6.5	0	no
D1	T2D	male	73	104	6.2	II	no
D2	T2D	male	76	16	6.6	II	no
D3	T2D	female	75	5	6.3	I	no
D4	T2D	male	73	23	6.9	I	no
D5	T2D	female	84	12	7.3	II	no
D6	T2D	male	91	36	6.4	II	no

Case codes follow the demographic table of the source publication [2]. The same 11 donors contributed the three cortical fractions, giving 33 arrays. Age and sex were included in the primary models. PMI, postmortem interval; NR, not reported; T2D, type 2 diabetes mellitus. PMI, tissue pH, Braak stage and dementia status are descriptive source characteristics and were not additional covariates in the primary model. Values were not imputed.

**Table A11:** GSE125387 sample assignments.

GEO Accession	GEO Sample Title	FPKM Column Label	Analysis Group
GSM3573174	db/m_1A	db_m_1A	db/m
GSM3573175	db/m_2A	db_m_2A	db/m
GSM3573176	db/m_3A	db_m_3A	db/m
GSM3573177	db/m_4A	db_m_4A	db/m
GSM3573178	db/m_5A	db_m_5A	db/m
GSM3573179	db/m_6A	db_m_6A	db/m
GSM3573180	db/m_7A	db_m_7A	db/m
GSM3573181	db/m_8A	db_m_8A	db/m
GSM3573182	db/m_9A	db_m_9A	db/m
GSM3573183	db/m_10A	db_m_10A	db/m
GSM3573184	db/m_11A	db_m_11A	db/m
GSM3573185	db/db_1A	db_db_1A	db/db
GSM3573186	db/db_2A	db_db_2A	db/db
GSM3573187	db/db_3A	db_db_3A	db/db
GSM3573188	db/db_4A	db_db_4A	db/db
GSM3573189	db/db_5A	db_db_5A	db/db
GSM3573190	db/db_6A	db_db_6A	db/db
GSM3573191	db/db_7A	db_db_7A	db/db
GSM3573192	db/db_8A	db_db_8A	db/db
GSM3573193	db/db_9A	db_db_9A	db/db
GSM3573194	db/db_10A	db_db_10A	db/db
GSM3573195	db/db_IF_1A	db_db_IF_1A	db/db + IF
GSM3573196	db/db_IF_2A	db_db_IF_2A	db/db + IF
GSM3573197	db/db_IF_4A	db_db_IF_4A	db/db + IF
GSM3573198	db/db_IF_5A	db_db_IF_5A	db/db + IF
GSM3573199	db/db_IF_6A	db_db_IF_6A	db/db + IF
GSM3573200	db/db_IF_7A	db_db_IF_7A	db/db + IF
GSM3573201	db/db_IF_8A	db_db_IF_8A	db/db + IF
GSM3573202	db/db_IF_9A	db_db_IF_9A	db/db + IF
GSM3573203	db/db_IF_10A	db_db_IF_10A	db/db + IF
GSM3573204	db/db_IF_11A	db_db_IF_11A	db/db + IF

All 31 labels agree between GEO, the deposited FPKM workbook and Supplementary Data S1 and S2 of the source publication [3]. Group counts are 11 db/m, 10 db/db and 10 db/db + IF. Slashes in GEO titles correspond to underscores in expression-column labels. The IF label numbered 3A is absent from both the deposit and the published tables; this numbering gap does not identify a reassignment. The source methods describe 10–11 mice per group for the multiomics cohort, whereas the source Figure 3a caption reports 11, 9 and 11. The methods also mention the loss of a db/db animal during water-maze testing, without linking it to a specific RNA-sequencing GSM record. No source-supported alternative assignment could be reconstructed, and the origin of the caption discrepancy remains unresolved.

**Table A12:** Gene universes and pathway eligibility after ribosomal exclusion.

Input	Original Genes	Lost Genes	Retained Genes	Original Eligible Sets	Reduced Eligible Sets
M-Hip	13,237	1	13,236	4282	4282
H-Astro	20,848	66	20,782	5198	5188
H-Neuron	20,848	66	20,782	5198	5188
H-Vasc	20,848	66	20,782	5198	5188
R-Hip	17,268	80	17,188	4859	4850
R-PFC	17,268	80	17,188	4859	4850
R-Str	17,268	80	17,188	4859	4850
KD whole brain	14,895	81	14,814	4923	4916
AD hippocampus	12,294	68	12,226	4148	4138

Exclusion used a strict native-symbol pattern for canonical cytosolic RPL/RPS genes; mitochondrial MRPL/MRPS and RPS6 kinase genes were retained. The original diabetic mouse input lost only *Rpl6l*, so this is not a comprehensive ribosomal-dependence test in that dataset. Eligibility and competitive background were recomputed. CAMERA applied to expression matrices at fixed ρ 0.01 is used for comparable eligibility counts. Tissue abbreviations are defined in Figure 1; KD, ketogenic diet; AD, APP/PS1.
